# The HIV Latency Reversal Agent HODHBt Enhances NK Cell Effector and Memory-Like Functions by Increasing Interleukin-15-Mediated STAT Activation

**DOI:** 10.1128/jvi.00372-22

**Published:** 2022-07-14

**Authors:** Amanda B. Macedo, Callie Levinger, Bryan N. Nguyen, Jonathan Richard, Mamta Gupta, Conrad Russell Y. Cruz, Andrés Finzi, Katherine B. Chiappinelli, Keith A. Crandall, Alberto Bosque

**Affiliations:** a Department of Microbiology, Immunology, & Tropical Medicine, The George Washington University, Washington, DC, USA; b Computational Biology Institute, Milken Institute School of Public Health, The George Washington University, Washington, DC, USA; c Department of Biostatistics & Bioinformatics, Milken Institute School of Public Health, The George Washington University, Washington, DC, USA; d Centre de Recherche du CHUM, Montreal, Quebec, Canada; e Département de Microbiologie, Infectiologie et Immunologie, Université de Montréal, Montreal, Quebec, Canada; f Department of Biochemistry & Molecular Medicine, School of Medicine & Health Sciences, The George Washington University, Washington, DC, USA; g GW Cancer Center, Washington, DC, USA; h Children’s National Medical Center, Washington, DC, USA; Emory University

**Keywords:** HIV, HODHBt, LRA, NK cells, STAT signaling, memory-like NK cells

## Abstract

Elimination of human immunodeficiency virus (HIV) reservoirs is a critical endpoint to eradicate HIV. One therapeutic intervention against latent HIV is “shock and kill.” This strategy is based on the transcriptional activation of latent HIV with a latency-reversing agent (LRA) with the consequent killing of the reactivated cell by either the cytopathic effect of HIV or the immune system. We have previously found that the small molecule 3-hydroxy-1,2,3-benzotriazin-4(3H)-one (HODHBt) acts as an LRA by increasing signal transducer and activator of transcription (STAT) factor activation mediated by interleukin-15 (IL-15) in cells isolated from aviremic participants. The IL-15 superagonist N-803 is currently under clinical investigation to eliminate latent reservoirs. IL-15 and N-803 share similar mechanisms of action by promoting the activation of STATs and have shown some promise in preclinical models directed toward HIV eradication. In this work, we evaluated the ability of HODHBt to enhance IL-15 signaling in natural killer (NK) cells and the biological consequences associated with increased STAT activation in NK cell effector and memory-like functions. We showed that HODHBt increased IL-15-mediated STAT phosphorylation in NK cells, resulting in increases in the secretion of CXCL-10 and interferon gamma (IFN-γ) and the expression of cytotoxic proteins, including granzyme B, granzyme A, perforin, granulysin, FASL, and TRAIL. This increased cytotoxic profile results in increased cytotoxicity against HIV-infected cells and different tumor cell lines. HODHBt also improved the generation of cytokine-induced memory-like NK cells. Overall, our data demonstrate that enhancing the magnitude of IL-15 signaling with HODHBt favors NK cell cytotoxicity and memory-like generation, and thus, targeting this pathway could be further explored for HIV cure interventions.

**IMPORTANCE** Several clinical trials targeting the HIV latent reservoir with LRAs have been completed. In spite of a lack of clinical benefit, they have been crucial to elucidate hurdles that “shock and kill” strategies have to overcome to promote an effective reduction of the latent reservoir to lead to a cure. These hurdles include low reactivation potential mediated by LRAs, the negative influence of some LRAs on the activity of natural killer and effector CD8 T cells, an increased resistance to apoptosis of latently infected cells, and an exhausted immune system due to chronic inflammation. To that end, finding therapeutic strategies that can overcome some of these challenges could improve the outcome of shock and kill strategies aimed at HIV eradication. Here, we show that the LRA HODHBt also improves IL-15-mediated NK cell effector and memory-like functions. As such, pharmacological enhancement of IL-15-mediated STAT activation can open new therapeutic avenues toward an HIV cure.

## INTRODUCTION

Human immunodeficiency virus (HIV) has caused more than 35 million deaths worldwide. Management of the disease requires the daily administration of a combination of antiretroviral (ART) drugs for the life of the infected individual. This is due to the presence of an intact and inducible latent reservoir of HIV that rebounds after discontinuation of ART therapy (1–3). Elimination of this latent reservoir is a critical endpoint to eradicate HIV. Therapeutic interventions against latent HIV have been mainly focused on “shock and kill” strategies (4–14). These strategies are based on the transcriptional activation of latent HIV with a latency-reversing agent (LRA) with the consequent killing of the reactivated cell by either the cytopathic effect of HIV or the immune system. Several clinical trials targeting the latent reservoir with LRAs have been completed (9, 15). In spite of a lack of clinical benefit of these initial trials, they have been crucial to elucidate hurdles that shock and kill strategies have to overcome to promote an effective reduction of the latent reservoir to lead to a cure. Among others, these hurdles include (i) low reactivation potential mediated by LRAs through promoting only transcription with low translation of HIV proteins (16), (ii) the negative influence of some LRAs on the activity of natural killer (NK) and effector CD8 T cells (17–19), (iii) an increased resistance to apoptosis of latently infected cells (20–23), and (iv) an exhausted immune system due to chronic inflammation (24, 25).

Interleukin-15 (IL-15) is a common gamma chain (γc) cytokine that promotes its biological effects through activation of the transcription factors signal transducer and activator of transcription 1 (STAT1), STAT3, and STAT5 (26). The IL-15 superagonist N-803 is a clinical candidate because it has enhanced biologic activity *in vivo* due to a longer serum half-life than recombinant IL-15, but they have the same mechanism of action (27, 28). Currently, there are five clinical trials involving the IL-15 superagonist N-803 in ART-suppressed people living with HIV (PLWH) (ClinicalTrials registration numbers NCT04808908, NCT04340596, NCT04505501, NCT05245292, and NCT02191098). The clinical trial with registration number NCT02191098 was a recently completed phase I study of N-803 that demonstrated N-803 to be safe to administer in PLWH (29). IL-15 and N-803 have been shown to (i) reactivate latent HIV both *ex vivo* and *in vivo* (18, 30, 31), (ii) enhance NK cell activity against HIV (32, 33), (iii) improve HIV-specific CD8 T cell responses (34), and (iv) promote the migration of NK and CD8 T cells to B cell follicles, a major compartment harboring latently infected cells (35, 36). However, the clinical benefit of IL-15 or N-803 can be hindered by the transient nature of cytokine signaling. Upon the binding of IL-15 to its receptor and activation of the Janus kinase (JAK)/STAT pathway, a series of negative feedback loops, including suppressor of cytokine signaling (SOCS) proteins, dephosphorylation by phosphatases, SUMOylation, and ubiquitination, reduce the transcriptional activity of STATs (37–42). We have recently published results showing that 3-hydroxy-1,2,3-benzotriazin-4(3H)-one (HODHBt) enhanced γc cytokine signaling in CD4 T cells by increasing the phosphorylation and transcriptional activity of STATs upon cytokine stimulation (43, 44). Our previous studies showed that HODHBt increased STAT5 phosphorylation (pSTAT5) after cytokine signaling concomitant with a reduction in SUMOylation, leading to increased STAT5 nuclear presence and transcriptional activity and binding of STAT5 to the HIV long terminal repeat (LTR) in primary CD4 T cells (43). HODHBt also enhanced the LRA activity of IL-15 in cells isolated from PLWH (44). As such, targeting this pathway may enhance the efficacy of using IL-15 or N-803 for cure approaches (35).

IL-15 is also critical for NK cell development, maturation, survival, proliferation, and cytotoxic function (45). In this work, we evaluated whether enhancing IL-15-mediated STAT activation with HODHBt could also improve NK cell effector and memory-like functions. We demonstrated that HODHBt enhanced IL-15-mediated NK cell activation, as demonstrated by increased expression of activation markers CD25 and CD69, as well as components of cytotoxic cell granules like granzyme B (GZM B), granzyme A (GZM A), perforin, and granulysin and death receptor ligands APO2L/TRAIL and CD95L/FASL. Furthermore, HODHBt enhanced IL-15-mediated secretion of interferon gamma (IFN-γ) and CXCL-10 by NK cells. These phenotypical changes were also associated with enhanced IL-15-mediated cytotoxicity against different tumor cell lines and HIV-infected CD4 T cells. Finally, IL-15, in combination with IL-12 and IL-18, has been shown to confer memory-like properties to NK cells. These properties include a quantitative increase in IFN-γ secretion upon restimulation (46). We found that the addition of HODHBt during the generation of memory-like NK cells led to enhanced IFN-γ production upon IL-12 and IL-15 recall while maintaining the ability to kill HIV-infected cells.

In conclusion, our results indicate that enhancing cytokine-induced STAT activation with HODHBt, or other small molecules targeting this pathway, may be a suitable pharmacological strategy to both reactivate latent HIV (43, 44) and enhance NK cell effector and memory-like functions and improve HIV cure strategies.

## RESULTS

### HODHBt enhances IL-15-mediated STAT phosphorylation and transcriptional activity in NK cells.

**FIG 1:**
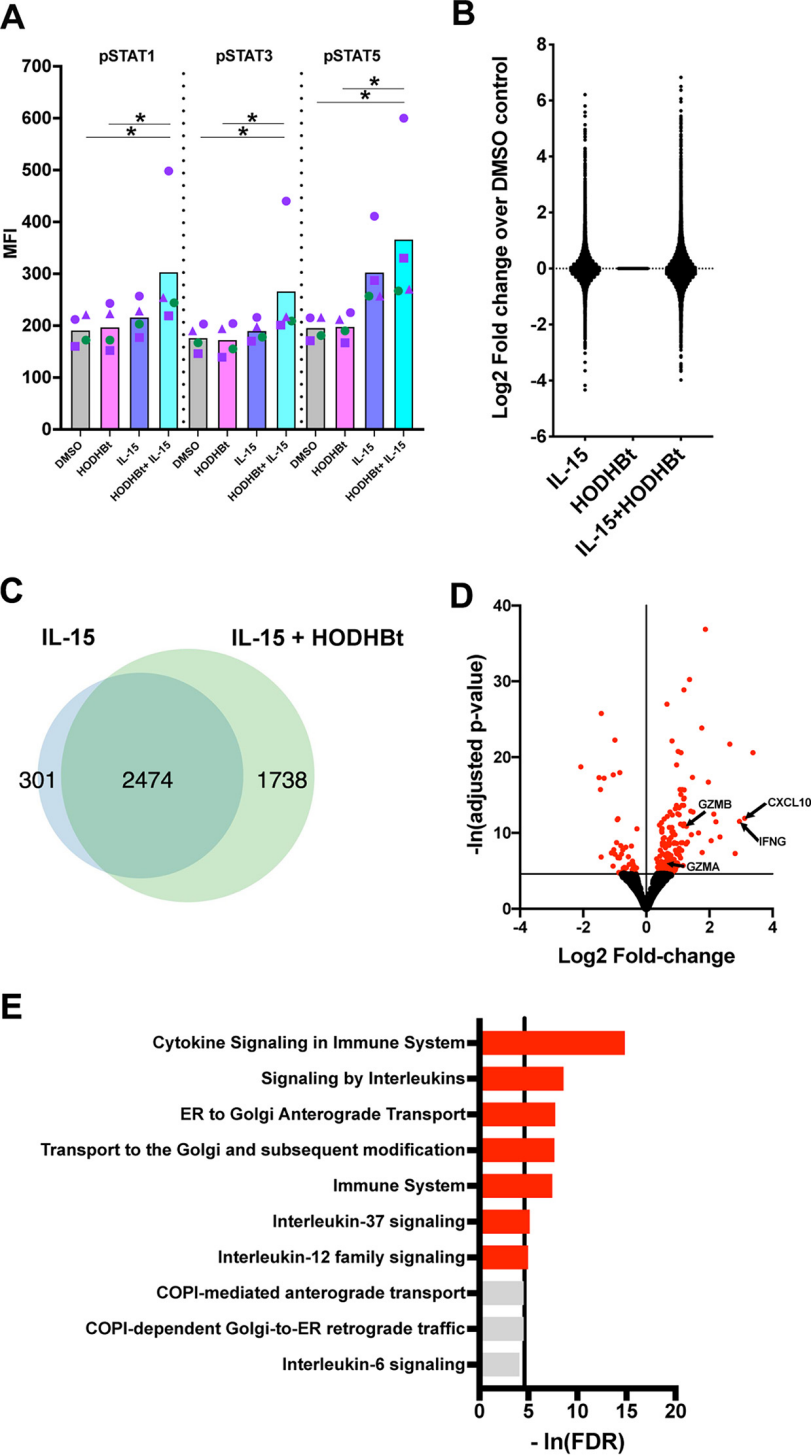
Biological effects of HODHBt in NK cells. (A) Levels of phosphorylated STAT1 (pSTAT1), pSTAT3, and pSTAT5 in NK cells from 4 donors. NK cells were treated with DMSO, IL-15 (100 ng/mL), HODHBt (100 μM), or a combination of both for 24 h. MFI, mean fluorescence intensity. *, *P* < 0.05, by Dunn’s multiple-comparison test. (B) Log_2_ fold changes over the expression in the DMSO control of genes regulated by IL-15, DMSO, or a combination of IL-15 and HODHBt as determined by RNA-Seq. (C) Venn diagram of genes differentially expressed between IL-15 and IL-15 plus HODHBt treatment compared to their expression in the DMSO control. (D) Volcano plot of genes differentially expressed between IL-15 and IL-15 plus HODHBt treatment. Genes with a change in expression having an adjusted *P* value of <0.01 are indicated in red. IFNG, IFN-γ. (E) Reactome pathway analysis of genes differentially expressed between IL-15 and IL-15 plus HODHBt treatment. Pathways with a false discovery rate (FDR) of <0.01 are indicated in red.

First, we confirmed whether HODHBt enhanced STAT phosphorylation upon cytokine stimulation on NK cells, as we have shown before for CD4 T cells (43, 44). For that, NK cells were isolated from peripheral blood mononuclear cells (PBMCs) and treated overnight with dimethyl sulfoxide (DMSO), recombinant human IL-15 (rhIL-15), HODHBt, or a combination of IL-15 plus HODHBt. After incubation, the levels of STAT5, STAT1, and STAT3 phosphorylation were analyzed by flow cytometry. Treatment with HODHBt alone did not substantially increase the levels of phosphorylation of any of the STATs analyzed (Fig. 1A). This agrees with our previous studies demonstrating that the activity of HODHBt is dependent upon a γc cytokine (43). rhIL-15 induced phosphorylation of STAT5 compared to its phosphorylation in the DMSO control. The combination of rhIL-15 plus HODHBt induced higher phosphorylation levels of the three STATs than did rhIL-15 alone (Fig. 1A). Next, we evaluated the transcriptional changes associated with increasing STAT phosphorylation with HODHBt. NK cells were treated overnight with DMSO, rhIL-15, HODHBt, or a combination of rhIL-15 plus HODHBt. Upon incubation, RNA was isolated and subjected to RNA sequencing (RNA-Seq) (Table S1 in the supplemental material). rhIL-15 and the combination of rhIL-15 plus HODHBt changed the transcription of 2,775 and 4,212 genes, respectively, compared to DMSO control (Fig. 1B and Table S2). Interestingly, HODHBt alone did not significantly change the transcription of any gene, in agreement with our previously published data showing minimal activity in the absence of a γc cytokine (Fig. 1B) (43). When comparing the differentially expressed (DE) genes induced by rhIL-15 and rhIL-15 plus HODHBt versus DMSO, 89.1% of the genes that were differentially expressed with rhIL-15 were also differentially expressed with rhIL-15 plus HODHBt. rhIL-15 plus HODHBt induced an additional 1,738 DE genes compared to rhIL-15 alone (Fig. 1C). We then compared the DE genes between rhIL-15 and rhIL-15 plus HODHBt. Only 202 were statistically significant, several of which are involved in the effector function of NK cells, including GZM B, GZM A, IFN-γ, and CXCL-10, and are well known downstream targets of cytokine signaling (Fig. 1D and Table S3) (47). Moreover, reactome pathway analysis indicated that the DE genes identified were involved in cytokine signaling pathways, confirming the specific role of HODHBt in enhancing cytokine signaling (Fig. 1E and Table S4).

**FIG 2:**
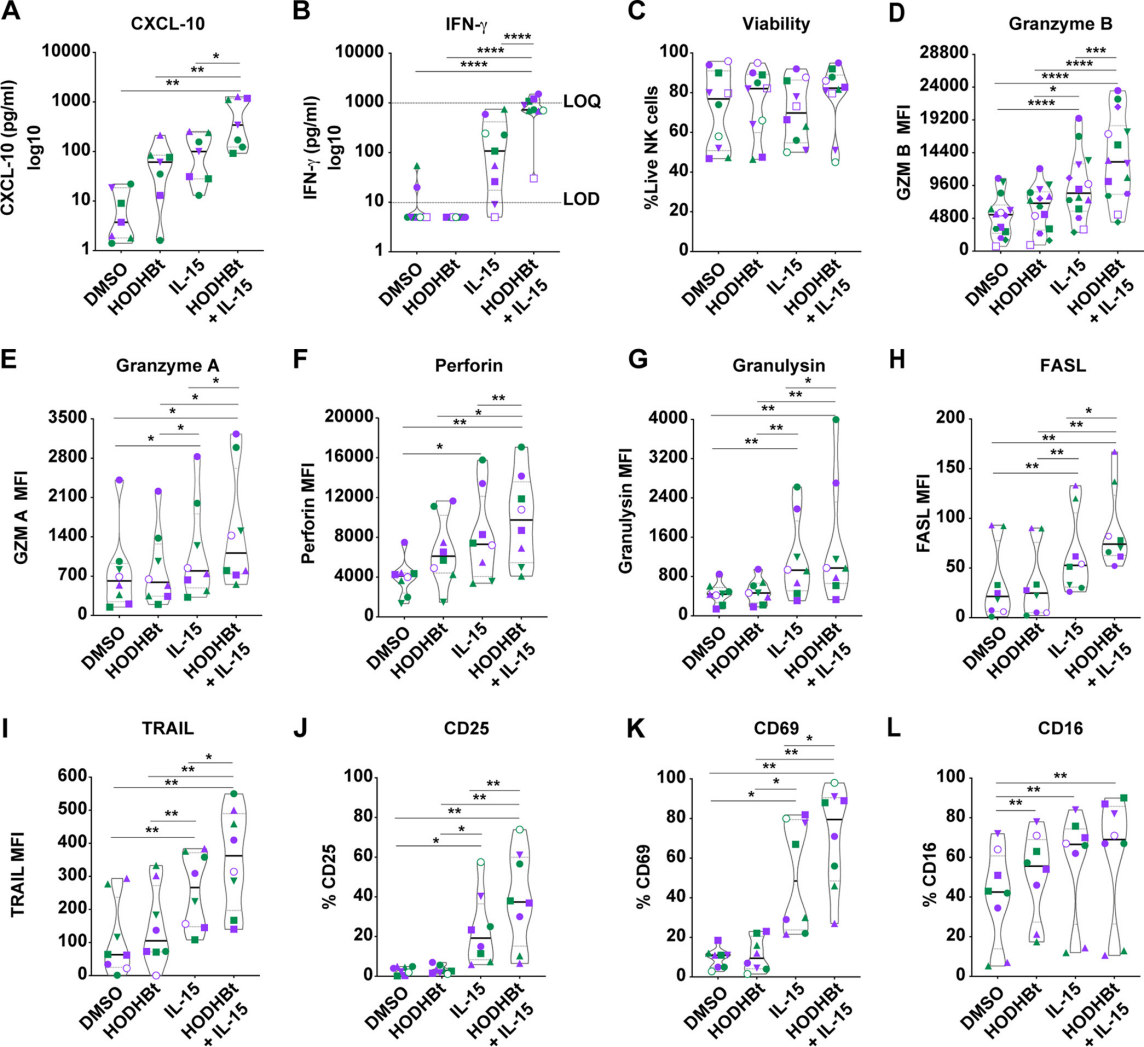
HODHBt enhances the cytotoxic profile of NK cells. (A, B) Levels of CXCL-10 (A) and IFN-γ (B) were quantified in cell culture supernatants after incubation of NK cells with the indicated treatments for 48 h. (C to L) Flow cytometric analysis of markers of viability (C), cytotoxicity (D to I), and activation (J to L) after incubation of NK cells with the indicated treatments for 48 h. Nonparametric Wilcoxon matched-pairs signed-rank test was used to calculate *P* values. Each symbol corresponds to a different donor. Purple symbols represent male and green female participants. *, *P* < 0.05; **, *P* < 0.01; ***, *P* < 0.001; ****, *P* < 0.0001, by analysis of variance (ANOVA) with Bonferroni correction.

We then confirmed the induction at the protein level of these four and other genes involved in NK cell effector function in cells isolated from additional 7 to 14 HIV-negative donor participants (Fig. 2). We first evaluated the secretion of the chemokine CXCL-10 and the cytokine IFN-γ by NK cells. The combination of rhIL-15 plus HODHBt enhanced the secretion of both compared to their secretion by rhIL-15 alone (Fig. 2A and B). Importantly, the presence of HODHBt was not associated with cell toxicity either alone or in combination with rhIL-15 (Fig. 2C). Flow cytometric analysis also confirmed enhanced protein expression of GZM B and GZM A under the rhIL-15 plus HODHBt condition compared to their expression with rhIL-15 alone (Fig. 2D and E). Next, we evaluated the expression of other proteins involved in NK cell cytotoxicity and activation. We observed increased protein expression of perforin, granulysin, FASL, and TRAIL, as well as the activation markers CD25 and CD69, but not CD16, under the rhIL-15 plus HODHBt condition compared to rhIL-15 alone (Fig. 2F to L). Interestingly, HODHBt alone was sufficient to increase the protein expression of CD16 compared to its expression in the DMSO control (Fig. 2L). However, this increase was not associated with increased gene transcription, based on our RNA-Seq analysis (Table S1).

**FIG 3:**
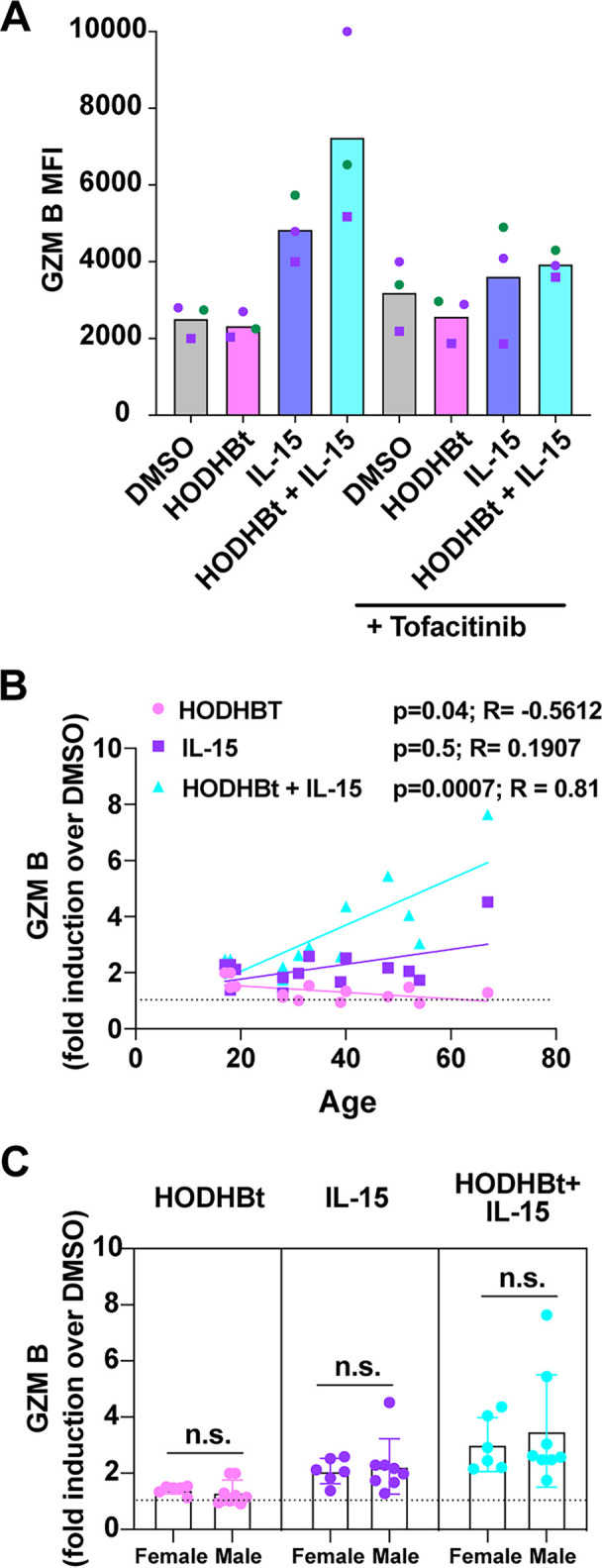
Influence of age and sex on granzyme B expression upon stimulation. (A) Levels of granzyme B after NK cells from 3 donors were incubated with the indicated treatments for 48 h in the presence of the JAK inhibitor tofacitinib. (B) Correlation between the induction of granzyme B expression (calculated as fold induction compared to that in the DMSO control) and age of the donors. (C) Comparison between the levels of expression of granzyme B by NK cells from male and female donors. Correlations were determined using the two-tailed nonparametric Spearman correlation coefficient. Mann-Whitney U test was used for comparisons between NK cells from female and male donors. n.s., not significant.

Next, we wanted to address whether the increase in protein expression mediated by HODHBt was associated with JAK/STAT activation or with a potential off-target effect of HODHBt. To that end, NK cells were treated overnight with DMSO, rhIL-15, HODHBt, or a combination of rhIL-15 plus HODHBt in the presence or absence of the JAK inhibitor tofacitinib. Tofacitinib completely abrogated GZM B induction by both rhIL-15 and rhIL-15 plus HODHBt (Fig. 3A). Finally, we observed a significant amount of variability in the response of NK cells to either rhIL-15 or the combination of rhIL-15 plus HODHBt. We decided to evaluate whether the biological sex or age of the donors could be a contributor in the variability observed in the induction of GZM B expression. Interestingly, the induction of GZM B (calculated as fold induction over that in the DMSO control) was significantly associated with age for rhIL-15 plus HODHBt (*P* = 0.0007, Spearman *r* = 0.81) (Fig. 3B). On the other hand, we did not observe any difference between cells from female and male donors regarding the ability of rhIL-15 or the combination of rhIL-15 plus HODHBt to induce the expression of GZM B (Fig. 3C). Overall, we confirmed that HODHBt enhanced rhIL-15-induced STAT phosphorylation and transcriptional activity on isolated NK cells, leading to the increased expression of several cytotoxic molecules important for NK cell function.

### HODHBt improves the ability of IL-15-activated NK cells to kill tumor cells and HIV-infected cells.

**FIG 4:**
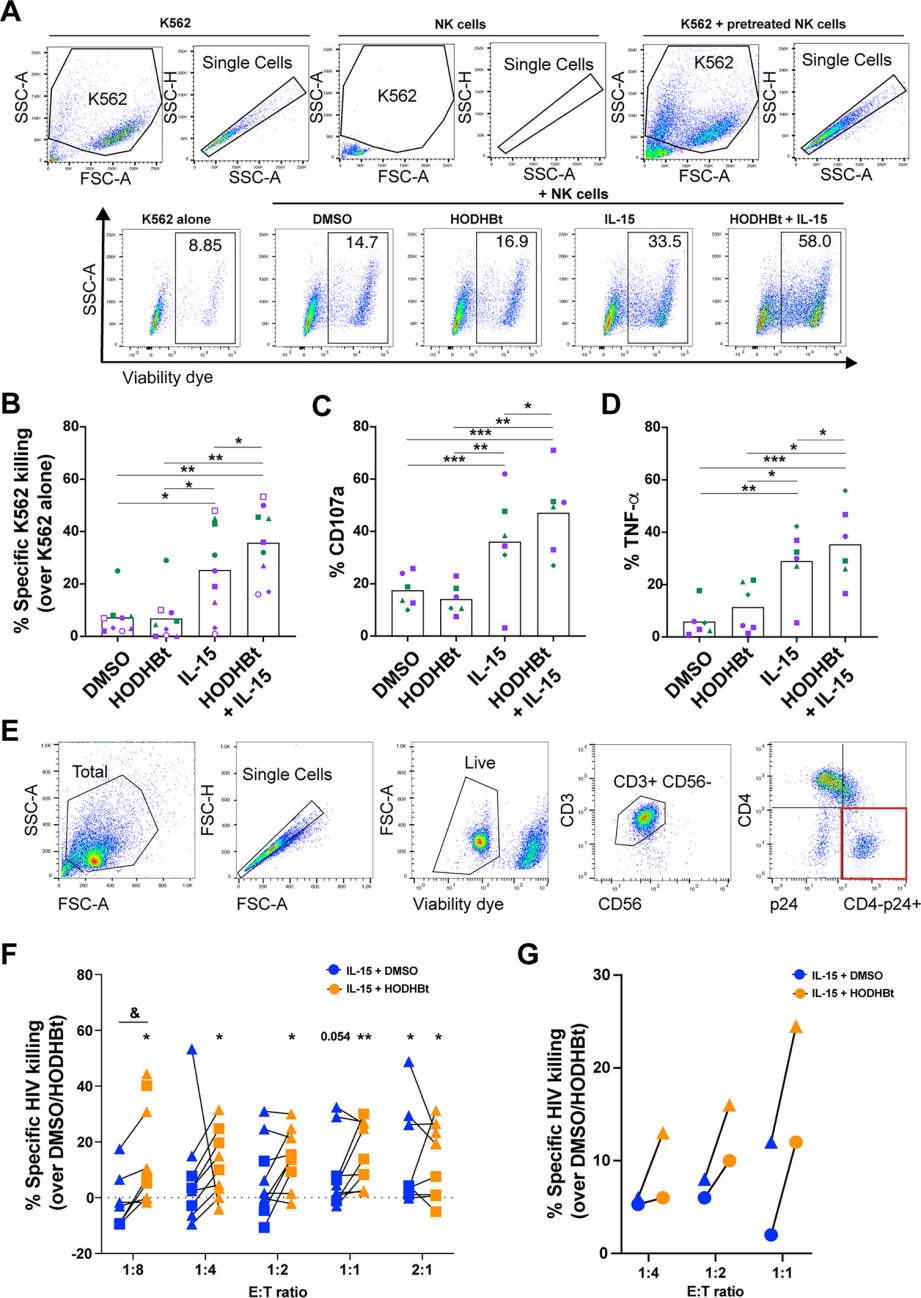
HODHBt enhances IL-15-mediated cytotoxicity of NK cells against HIV-infected cells. (A) Representative flow cytometry gating strategy from one experiment, indicating selection of cells to measure cell death of K562 cells cultured alone or in the presence of NK cells preincubated with the indicated treatments for 24 h at a 1:1 effector-to-target (E/T) ratio. SSC, side scatter; FSC, forward scatter. (B) K562 cell death mediated by pretreated NK cells from 6 male (purple) and 3 female (green) participants. (C, D) Surface expression of CD107a (C) and intracellular TNF-α (D) in NK cells cocultured with K562 cells. Purple symbols represent male and green female participants. (E) Representative flow cytometry gating strategy from one experiment indicating selection of CD3-positive cells to measure the reduction of p24^+^ CD4 T cells in the absence or presence of pretreated NK cells. (F) Percentages of specific HIV-infected-cell killing compared to the results for the DMSO or HODHBt control in overnight cocultures of NK cells pretreated with rhIL-15 plus DMSO or HODHBt and HIV-infected CD4 T cells at different E/T ratios. Square symbols represent male and triangles female participants. (G) Specific killing of CH058-infected CD4 T cells by pretreated NK cells at different E/T ratios. &, *P* < 0.05, by two-tailed Wilcoxon matched-pairs signed-rank test, comparing IL-15 plus DMSO with IL-15 plus HODHBt treatment; *, *P* < 0.05; **, *P* < 0.01, by Wilcoxon signed-rank test, to evaluate killing compared to the results for the DMSO or HODHBt control condition.

Based on the previous results demonstrating that enhancing STAT activation with HODHBt increased the expression of several proteins involved in NK cell cytotoxicity, we were interested to test whether HODHBt also increased the ability of IL-15-activated NK cells to kill different cancer cell lines, including both hematologic and solid tumors, as well as HIV-infected cells. We first tested whether HODHBt enhanced the ability of IL-15-activated NK cells to kill the erythroleukemia K562 cell line, which lacks major histocompatibility complex (MHC) class I, making these cells a target for NK cells (48). NK cells pretreated with either DMSO or HODHBt had low capacity to kill K562 cells at a 1:1 effector-to-target (E/T) ratio (Fig. 4A and B). Pretreatment of NK cells with rhIL-15 alone induced higher target killing than DMSO or HODHBt alone, while NK cells treated with the combination of rhIL-15 plus HODHBt had the highest killing ability of the four groups (Fig. 4A and B). We then used the Bliss independence model to evaluate whether the enhanced killing observed with the treatment combination was synergistic (49). The Bliss model is based on probability theory and assumes that when two drugs act through independent mechanisms, the expected (fa*_xy_*_,_*_e_*) combinatorial effect should be the sum of the two fractional responses (fa) minus their product [(fa*_x_* + fa*_y_*) − (fa*_x_* × fa*_y_*)]. The interaction of each combination is described by the difference between the observed (*o*) and the expected (e) response (Δfa*_xy_* = fa*_xy_*_,_*_o_* − fa*_xy_*_,_*_e_*). Bliss independence analysis yields synergistic (Δfa*_xy_* > 0), independent (Δfa*_xy_* = 0), or antagonistic (Δfa*_xy_* < 0) combinatorial interactions (49). In this case, the fraction of cell death induced by either HODHBt-treated or rhIL-15-treated NK cells alone was used to calculate fa*_xy_*_,_*_e_* and compared with the fraction of cell death induced by NK cells treated with a combination of both (fa*_xy_*,*_o_*). This analysis demonstrated that the combination of rhIL-15 with HODHBt was synergistic (Δfa*_xy_* = 0.05). The increased NK cell killing capacity mediated by the combination of rhIL-15 with HODHBt was also concomitant with an increase in NK cell degranulation and cytokine release, as measured by CD107a expression (Fig. 4C) and tumor necrosis factor alpha (TNF-α) production (Fig. 4D), respectively. Next, we were interested in testing whether HODHBt would increase NK cell killing of HIV-infected CD4 T cells. As HODHBt has been shown to reactivate latent HIV (43, 44), it will be important to address whether targeting this pathway can also enhance the ability of NK cells to kill HIV-infected cells. Furthermore, it is known that HIV-infected CD4 T cells are naturally resistant to NK cell killing (50–52). We observed that coculturing IL-15-activated NK cells with HIV_NL43_-infected autologous CD4 T cells induced higher killing of HIV-infected CD4 T cells than coculturing them with DMSO-treated NK cells at 1:1 and 2:1 E/T ratios, similar to results previously published by others (Fig. 4E and F) (32). The addition of HODHBt to rhIL-15 enhanced the killing of infected cells over that of HODHBt-treated NK cells at all E/T ratios tested and over that of rhIL-15 alone at a 1:8 E/T ratio (Fig. 4F). A similar increase was observed using the transmitted/founder (TF) virus CH058 (Fig. 4G).

**FIG 5:**
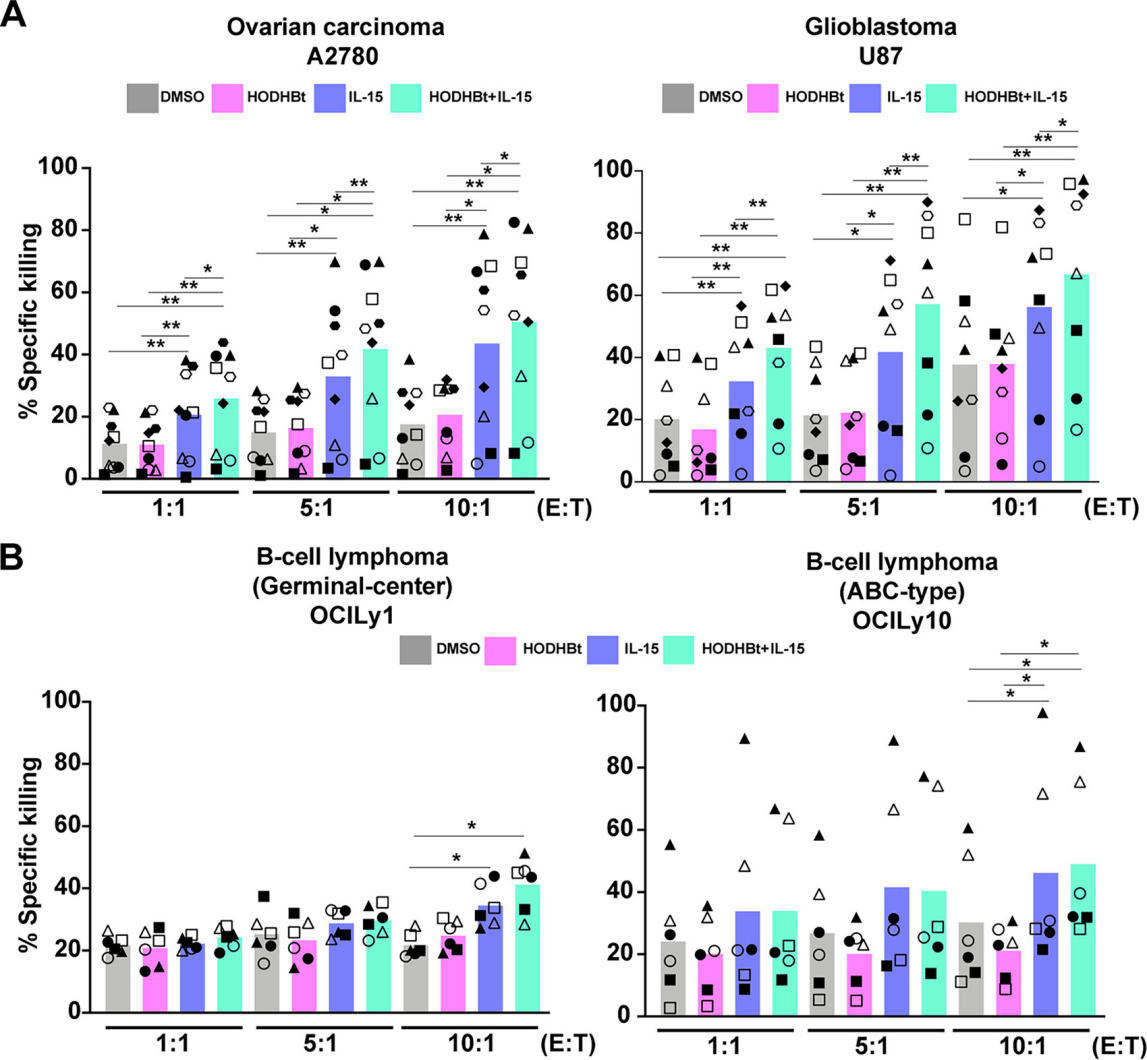
HODHBt enhances IL-15-mediated cytotoxicity of NK cells against cancer cell lines. Percentages of A2780 and U877 (A) and OCILy1 and OCILy10 (B) target cell lysis after incubation with pretreated NK cells at different E/T ratios. Black symbols represent male and white female participants. *, *P* < 0.05; **, *P* < 0.01, by two-tailed Wilcoxon matched-pairs signed-rank test.

Next, we extended our studies to four other transformed cell lines, including the human ovarian carcinoma cell line A2780, the glioblastoma cell line U87, the germinal center cell line OCILy1, and the B cell lymphoma cell line OCILy10. Treatment with rhIL-15 enhanced the NK cell killing of A2780 and U87 cells at different E/T ratios (Fig. 5A). As for K562 cells, HODHBt alone did not increase NK cells’ cytotoxicity, but it did enhance the ability of IL-15-treated NK cells to kill both tumor cell lines (Fig. 5A). However, the ability of rhIL-15 or the combination of rhIL15 plus HODHBt to increase the killing capacity of NK cells was not observed in all cancer types studied. Neither rhIL-15 nor the combination substantially increased the ability of NK cells to kill the germinal center OCILy1 or B cell lymphoma OCILy10 cell lines in comparison to the killing ability of untreated NK cells (Fig. 5B). In conclusion, we demonstrated that, upon rhIL-15 stimulation, HODHBt could enhance the killing capacity of NK cells on HIV-infected CD4 T cells and different cancer cell models.

### Long-term exposure to HODHBt does not cause exhaustion of NK cells.

**FIG 6:**
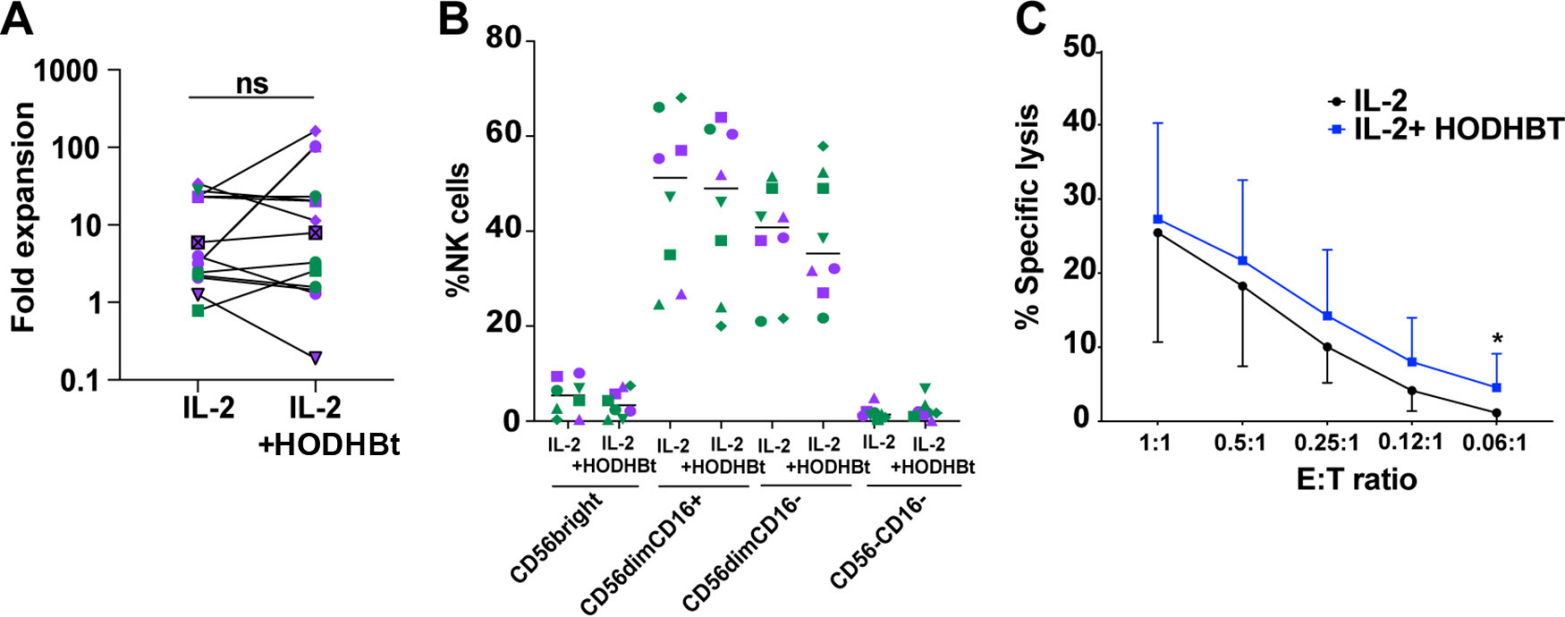
Long-term exposure to HODHBt does not affect NK cell proliferation, phenotype, or cytotoxicity. (A) Two-week fold expansion of NK cells with either rhIL-2 or rhIL-2 plus HODHBt. (B) Percentages of 4 different NK cell subsets based on CD56 and CD16 expression after 14 days of culture. Purple symbols represent male and green female participants. (C) Killing of K562 cells by expanded NK cells at different E/T ratios. *, *P* < 0.05, by 2-tailed Wilcoxon matched-pairs signed-rank test, to compare stimuli.

Higher activation of NK cells with HODHBt could lead to anergy and alteration of NK cell killing ability. To evaluate whether long-term exposure to HODHBt and chronic STAT activation could be detrimental for NK cell function, we used a commercial medium for NK cell expansion that requires the addition of the γc cytokine IL-2. IL-2, like IL-15, activates STAT5, -3, and -1, and we have shown that HODHBt enhances the activation of STATs mediated by IL-2 (43). We followed NK cell expansion with this protocol for 14 days. The presence of HODHBt through the 14-day period did not alter the expansion of NK cells or their phenotype based on CD56 and CD16 expression (Fig. 6A and B). Finally, we evaluated whether long-term exposure to HODHBt would alter the killing capacity of NK cells, using the erythroblastoma cell line K562 as the target. We did observe that, in general, expansion in the presence of HODHBt increased the killing capacity of NK cells, albeit it was only statistically significant at the lowest E/T ratio tested (0.06:1), possibly due to the high variability between participants (Fig. 6C). These results suggested that long-term exposure of NK cells to HODHBt and continued STAT activation did not induce anergy and might have increased the overall killing capacity of NK cells.

### HODHBt increases the generation of CIML NK cells.

**FIG 7:**
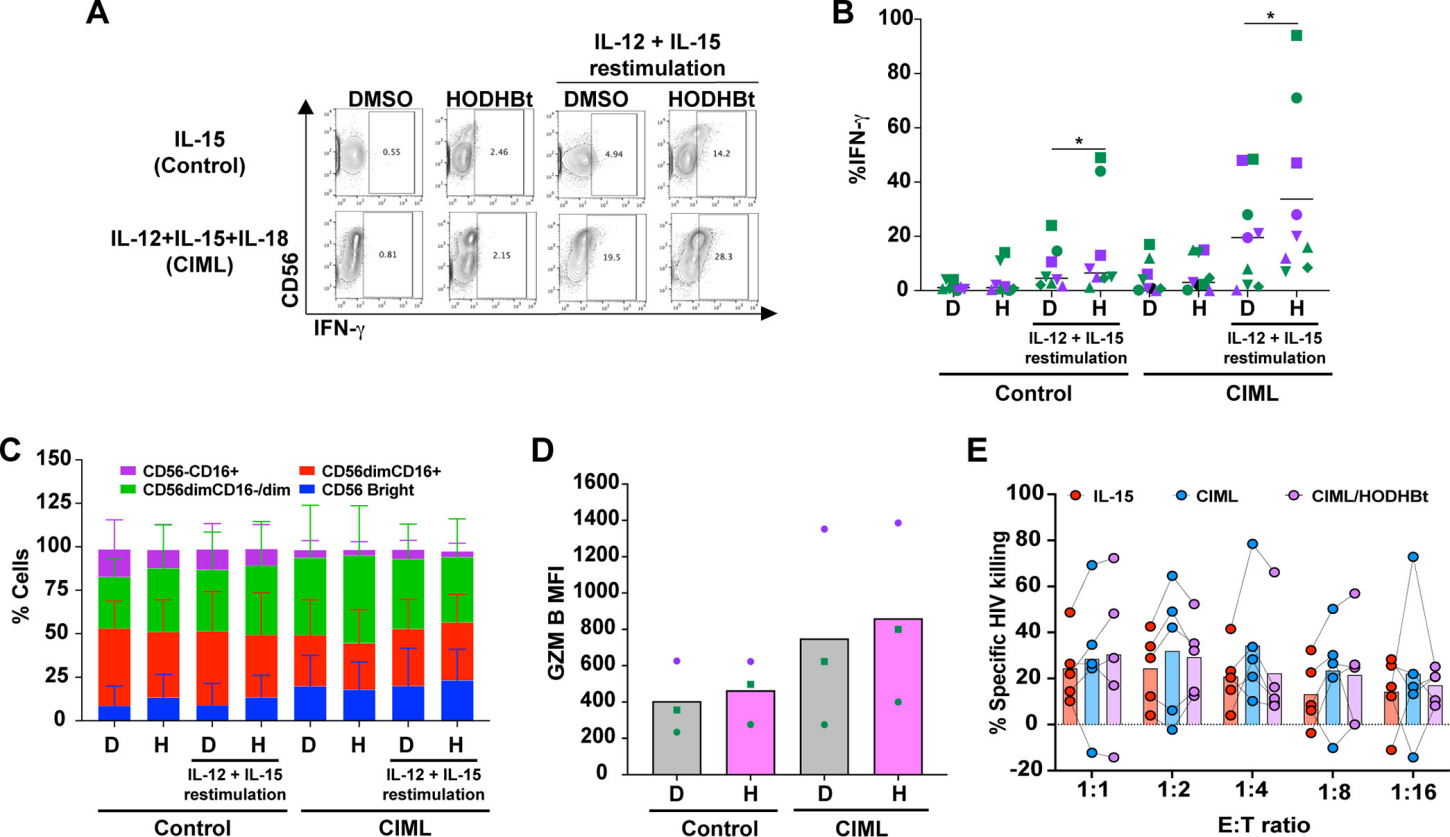
*In vitro* generation of human CIML NK cells with HODHBt results in enhanced memory response upon recall. (A) Representative flow cytometry plots denotating NK cells preactivated with either rhIL-15 (Control) or a combination of rhIL-12, rhIL-15, and rhIL-18 (CIML) in the presence of DMSO or HODHBt expressing IFN-γ with and without recall with rhIL-12 and rhIL-15. (B) Intracellular IFN-γ after recall in NK cells from 4 male (purple) and 5 female (green) participants. (C) Comparisons of NK cell subsets based on CD56 and CD16 expression in cells from 3 male and 4 female participants. (D) Levels of GZM B in control or CIML NK cells from 1 male and 2 female participants. (E) Percentages of specific killing of HIV-infected CD4 T cells in overnight cocultures of control or CIML NK cells generated with either DMSO or HODHBt and infected CD4 T cells at different E/T ratios. D, DMSO; H, HODHBt. *, *P* < 0.05, by 2-tailed Wilcoxon matched-pairs signed-rank test.

NK cells have been shown to have “adaptive” or “memory-like” properties (53–55). These properties include a quantitative and qualitative increase in effector response upon restimulation, characterized by enhanced IFN-γ production (56). In humans, memory-like NK cell differentiation can be induced by cytokine stimulation with a combination of rhIL-12, rhIL-18, and rhIL-15, and those cells are called cytokine-induced memory-like (CIML) natural killer cells (56, 57). CIML NK cells are characterized by an epigenetic remodeling of the IFN-γ locus, including reduced DNA methylation and enhanced IFN-γ upon restimulation in response to rhIL-12 and rhIL-15, and enhanced survival *in vivo* (56, 57). Since IL-12 mediates STAT4 activation and IL-15 activates STAT5 (53, 58), we decided to investigate whether HODHBt could increase the generation of CIML NK cells by enhancing STAT activation. First, to evaluate whether HODHBt enhanced CIML NK cell generation, we preactivated NK cells using rhIL-15 (1 ng/mL) (control) or rhIL-12 (10 ng/mL), rhIL-18 (50 ng/mL), and rhIL-15 (1 ng/mL) (CIML) in the presence of DMSO or HODHBt. On the next day, cells were washed and cultured in low concentrations of rhIL-15 (1 ng/mL) without additional DMSO or HODHBt. After 7 days, NK cells were restimulated with 100 ng/mL of rhIL-12 and rhIL-15, and intracellular production of IFN-γ was measured by flow cytometry (Fig. 7A). In general, NK cells preactivated with IL-15 alone (control) produced less IFN-γ upon cytokine recall than CIML NK cells regardless of whether cells were preactivated in the presence of DMSO or HODHBt (Fig. 7B). Interestingly, HODHBt increased the percentage of cells producing IFN-γ upon cytokine recall for both control and CIML NK cells (Fig. 7B). Compared with the IL-15 control, CIML NK cell stimulation was associated with an increase in the CD56^bright^ population (Fig. 7C) and with an increase in the levels of GZM B (Fig. 7D), but the presence of HODHBt did not alter the overall phenotype or GZM B expression. Increased cytokine production by NK cells has been associated with a reduction in their cytotoxic potential (59, 60). The generation of CIML NK cells in the presence or absence of HODHBt did not interfere with the killing of autologous HIV_AD8_-infected CD4 T cells compared with their killing by the IL-15 control (Fig. 7E). In conclusion, increasing STAT activation with HODHBt enhanced the generation of CIML NK cells *in vitro*, indicating that the magnitude of the STAT activation could be a factor contributing to the generation of memory-like responses in NK cells, albeit the CIML phenotype did not seem to contribute to an enhanced ability to kill HIV-infected cells.

## DISCUSSION

In this study, we tested whether enhancing STAT activation with the LRA HODHBt could enhance cytokine-mediated NK cell cytotoxicity and memory-like generation *in vitro*. We observed that HODHBt increased the phosphorylation of STAT5, STAT3, and STAT1 in NK cells treated with IL-15. This increase in phosphorylation by HODHBt was associated with higher STAT transcriptional activation, leading to an increased cytotoxic profile phenotype. This was demonstrated by increased expression of activation markers (CD25 and CD69), cytotoxic proteases (GZM A, GZM B, perforin, and granulysin), death receptor ligands (TRAIL and FASL), and cytokine production (IFN-γ and CXCL-10). Moreover, HODHBt enhanced the ability of IL-15-activated NK cells to kill HIV-infected CD4 T cells and different tumor cell lines, including chronic myelogenous leukemia, ovarian carcinoma, and glioblastoma cell lines. Finally, HODHBt also enhanced the generation of CIML NK cells.

NK cells are part of the innate immune system and play an important role in controlling HIV infection. NK cells are important in the control of simian immunodeficiency virus (SIV) replication in the B cell follicles of African green monkeys (AGMs) (61). Furthermore, a recent vaccination study using a pentavalent HIV vaccine that builds upon the RV144 study revealed a decrease in acquisition of simian-human immunodeficiency virus (SHIV) due to an increase in the activity of NK cells (62, 63). Finally, Saez-Cirion et al. showed stronger NK cell responses in posttreatment controllers than in noncontrollers from the VISCONTI study (64). IL-15, through the activation of STATs, is critical for NK cell development, maturation, survival, proliferation, and cytotoxic function (45, 65). In fact, STAT5 has been proposed as the master transcriptional regulator and plays a role in NK cell maturation, survival, and cytotoxicity (66–68). Using an *in vitro* primary model, we observed that IL-15 plus HODHBt enhanced the killing of HIV-infected cells compared to the killing with IL-15 alone. As such, our results suggested that pharmacologic enhancement of IL-15-mediated STAT activation could improve NK cell activity against HIV. For example, robust NK cell effector functions could lead to better control of HIV replication (62–64). Chronic HIV-1 infection also leads to pathological changes in NK cells, including defective functionality, and control of viremia with ART has been reported to restore some but not all NK cell activity in PLWH (69–72). Those impairments include lower NK cell cytotoxicity and IFN-γ production, especially in ART patients with incomplete recovery of CD4 counts (73). Moreover, HIV infection even in the context of ART has been associated with higher risks of developing certain types of cancer (74, 75). As such, identifying pathways that can enhance NK cell effector function may benefit PLWH not only to control HIV infection but also to reduce the risk for cancer development. Furthermore, there are five clinical trials involving the use of the IL-15 superagonist N-803 in ART-suppressed PLWH to promote the elimination of latent HIV. Although HODHBt is not currently a clinical candidate, our studies suggest that enhancing IL-15-mediated STAT activation could be a potential mechanism to synergize with N-803 to reactivate latent HIV and rescue NK cell effector activity. Further efforts will be needed to develop this class of molecules into a clinical candidate or to identify novel compounds that target the same pathway.

Modulation of STAT activation with HODHBt could have an immediate application. The clinical evaluation of NK cells for use in adoptive cell immunotherapies has increased in the last decade (76–81). NK cells are an alternative to T cell immunotherapies because they preferentially target transformed cells, without the need for prior sensitization or known antigens. Also, NK cells are not thought to elicit graft-versus-host disease, and “universal donor” off-the-shelf NK cells are being developed. Despite their promise, chronic exposure to cancer cells leads to impairment of NK cell function. For this reason, multiple strategies have been developed to boost the antitumor effect of NK cells and abolish tumor resistance. Some examples include adoptive transfer of NK cells after *ex vivo* activation and expansion, restoration of NK cell function using immune checkpoint inhibitors and monoclonal antibodies, and cytokine treatment. In this work, we have performed a proof-of-concept study to demonstrate that enhancing IL-15-mediated STAT activation using the small molecule HODHBt can improve NK cell responses. We have observed that HODHBt improved NK cell-mediated killing of several cancer cell lines representative of erythroblastoma, ovarian cancer, and glioblastoma compared to treatment with IL-15 alone. However, we have observed that B cell lines are less sensitive to enhanced NK cell killing by IL-15 alone or in combination with HODHBt. These data indicate that modulating the STAT pathway may not be sufficient to enhance NK cell killing of some cancer types due to intrinsic cell resistance and that additional strategies will be needed. It will be interesting to test whether combination therapy of HODHBt with other current strategies against B cell lymphomas, such as NK cells expressing chimeric antigen receptors (CARs) against myeloid antigens, could overcome this resistance (82).

Adoptive NK cell therapy exhibits promise for both cancer and HIV therapy, and the development of additional robust methods to expand large numbers of highly effective NK cells is an important area of research. In this study, we tested the effect of HODHBt supplementation in a commercially available NK cell expansion medium with IL-2. The supplementation did not affect the proliferation rate nor the phenotype of the NK cells but increased the ability of NK cells to kill K562. It would be interesting to test whether HODHBt would enhance expansion using different proliferation protocols and other cytokines (83–85).

Although NK cells have traditionally been classified as cells of the innate immune system, they have been demonstrated to have memory features, mounting responses upon recall (55, 86, 87). In the context of HIV, preexisting memory-like NK cells can control viremia in primary infection (88, 89). Furthermore, SIV-infected macaques but not uninfected macaques have memory-like NK cells able to kill in an antigen-specific manner dendritic cells pulsed with either Gag or Env peptides (90). A recent study by Wang et al. has identified that ongoing viral replication in HIV-infected individuals increases the proportion of memory-like NK cells (91). In addition, it has been demonstrated that NK cells preactivated *in vitro* with rhIL-12, rhIL-15, and rhIL-18 (so called CIML NK cells) produced higher levels of IFN-γ upon cytokine restimulation (56, 57, 92). In this work, we were able to demonstrate that HODHBt also enhanced the generation of CIML NK cells, based on a higher IFN-γ recall response, without detrimental effects on their killing capacity for HIV-infected cells. This suggests that the magnitude of STAT activation could be a contributing factor molding the development of a memory-like phenotype in NK cells. Further studies are warranted to delineate the specific mechanisms involved in this process.

Our studies are not without caveats. We have observed a large variation among donors in the response to IL-15, as well as to IL-15 plus HODHBt. Multiple subsets of NK cells have been identified, and how IL-15 or HODHBt affects all these subsets is largely unknown (93). We have restricted our studies to identified changes in the main NK cell subsets that are characterized by the expression of CD56 and CD16. We did not observe a significant change in the proportion of any of these subsets upon HODHBt treatment (Fig. 6 and 7). Further studies using single-cell RNA-Seq will be warranted to fully understand the phenotypic changes associated with HODHBt treatment and enhancement of STAT activity. Besides natural cytotoxicity, NK cells can exert their cytotoxicity by antibody-dependent cell cytotoxicity (ADCC). ADCC requires the expression of CD16 in NK cells (94). HODHBt did not alter the expression of CD16 in combination with IL-15 (Fig. 2). However, further studies will be warranted to evaluate whether HODHBt can influence ADCC.

In conclusion, our data show that enhancing cytokine-induced STAT activation with HODHBt significantly increases the NK cell cytotoxicity phenotype and function and the generation of CIML NK cells. HODHBt could be further exploited for cell adoptive immunotherapeutic approaches using NK cells against cancer and HIV. Furthermore, the development of clinically relevant compounds targeting this pathway could be an attractive area of research to enhance *in vivo* the effector function of NK cells for HIV cure approaches or against different malignancies or other infectious diseases.

## MATERIALS AND METHODS

### Cell lines and reagents.

rhIL-2 and rhIL-15 were provided by the BRB/NCI Preclinical Repository. rhIL-12 was obtained from PeproTech and rhIL-18 from R&D systems. HODHBt was purchased from AK Scientific. K562-green fluorescent protein (GFP) (ATCC CCL-242-GFP) cell culture was maintained in Dulbecco’s modified Eagle medium (DMEM) supplemented with 10% fetal bovine serum (FBS), glutamine, and penicillin-streptomycin. A2780 cell culture was maintained in RPMI 1640 medium supplemented with 10% FBS, glutamine, and penicillin-streptomycin. U87 cell culture was maintained in DMEM supplemented with 10% heat-inactivated FBS, glutamine, OCILy1 and OCILy10 cell cultures were maintained in Iscove’s modified Dulbecco’s medium (IMDM) medium supplemented with 10% human serum, glutamine, and penicillin-streptomycin. Nelfinavir and raltegravir were obtained through the AIDS Research and Reference Reagent Program, Division of AIDS, NIAID, and HIV-1_NL4-3_ from Malcolm Martin.

### Primary cell culture.

Primary NK cells and naive CD4 T cells were isolated from PBMCs by negative selection with cell-type-specific EasySep (Stem Cell Technologies) according to the manufacturer’s protocol. The purity of isolated NK cells (CD3^−^ CD56^+^ CD16^−/+^) was verified by flow cytometric analysis. NK cells were stimulated with DMSO, 100 ng/mL rhIL-15, 100 μM HODHBt, or rhIL-15 plus HODHBt overnight or at the times indicated in the figure legends. For experiments using the JAK inhibitor tofacitinib, NK cells were pretreated with 30 ng/mL of tofacitinib (Cayman Chemical) for 1 h prior to stimulation. For expansion experiments, enriched NK cells were cultured in MACS medium (order no. 130-114-429) from Miltenyi Biotech for 14 days (with 500 IU/ML of rhIL-2 in the absence or presence of 100 μM HODHBT) following the manufacture’s protocol. For the CIML NK cell experiments, NK cells were plated at 2 to 5 × 10^6^ cells/mL and preactivated for 16 h using rhIL-12 (10 ng/mL), rhIL-18 (50 ng/mL), and rhIL-15 (1 ng/mL) in the presence of HODHBt or of DMSO as a control and cultured in complete RPMI 1640 medium containing 10% human AB serum (Sigma-Aldrich). As a control, NK cells with only rhIL-15 were plated in medium in the presence of DMSO or HODHBt. On the next day, cells were washed, counted, and replated with rhIL-15 (1 ng/mL) to support survival, with 50% of the medium being replaced every 2 or 3 days along with fresh cytokine. After 7 days, cells were harvested, washed, and restimulated with IL-12 (10 ng/mL) plus IL-15 (100 ng/mL) for 6 h in a 96-well round-bottom plate. Protein transport inhibitor cocktail (eBioscience) was added after 1 h, and cells were stained for surface NK cell markers (CD56 and CD16) and intracellular IFN-γ (Cytofix/Cytoperm; BD Biosciences).

### RNA-Seq analysis.

RNA from 5 to 10 million NK cells treated overnight under the indicated conditions was extracted using an RNeasy plus kit (Qiagen). Samples were prepared for Illumina sequencing following the manufacturer’s protocol using the TruSeq stranded total RNA library prep kit with rRNA depletion using the Ribo-Zero human/mouse/rat kit. First-strand synthesis was completed using SuperScript III (catalog no. 18080044; Thermo Fisher), with an extension temperature of 50°C. Sequencing was performed using a NextSeq high-output kit, version 2.5 (150 cycles) (catalog no. 20024907; Illumina) on a NextSeq 500, with single indexing. The resulting sequence data were quality controlled using FastQC (https://www.bioinformatics.babraham.ac.uk/projects/fastqc/) and MultiQC (95). Low-quality reads were trimmed using Trimmomatic (96). Differential gene expression was calculated with DESeq2 (97). Reactome pathway analysis was performed using analysis tools at https://reactome.org (98).

### Flow cytometry.

To evaluate STAT phosphorylation, cells were stained with antibodies against pSTAT1(Y701) (clone 4a), pSTAT3(Y705) (clone 4/P), and pSTAT5(Y694) (clone 47) (BD Biosciences) as previously described (43).

To evaluate NK cell activation, cells were washed in phosphate-buffered saline (PBS), incubated with LIVE/DEAD fixable aqua stain (Invitrogen) for 10 min, and washed, and then anti-human antibodies to the following proteins were used for surface staining: CD3-BV786 (clone SP34-2, catalog no. 563800; BD), CD56-BV605 (clone HCD56, catalog no. 318334; Biolegend), CD16-fluorescein isothiocyanate (FITC) (clone 3G8, catalog no. 555406; BD), CD69-allophycocyanin (APC)-Cy7 (clone FN50, catalog no. 310914; Biolegend), and CD25-phycoerythrin (PE) (clone BC96, catalog no. 12-0259-42; eBiosciences). To evaluate cytotoxic profiles, cells were washed in PBS, incubated with LIVE/DEAD fixable aqua stain (Invitrogen) for 10 min, and washed, and then anti-human antibodies to the following proteins were used for surface staining: CD3-BV786 (clone SP34-2, catalog no. 563800; BD) and CD56-BV605 (clone HCD56, catalog no. 318334; Biolegend). After surface staining, NK cells were fixed and permeabilized with Cytofix/Cytoperm buffer (catalog no. 554722; BD Biosciences), followed by intracellular staining with antibodies to granzyme B-AF700 (clone GB11, catalog no. 560213; BD Biosciences), granzyme A-PE-Cy7 (clone CB9, catalog no. 507221; BD Biosciences), perforin-FITC (clone dG9, catalog no. 308104; Biolegend), granulysin-PE (clone DH2, catalog no. 308104; Biolegend), TRAIL-APC (clone N2B2, catalog no. 109310; Biolegend), and FASL-BV421 (clone DX2, catalog no. 11-0959-42; eBiosciences). For experiments with HIV-infected CD4 T cells, cells were stained using LIVE/DEAD fixable aqua stain (Invitrogen) and antibodies to the following proteins: CD3-BV786 (clone SP34-2, catalog no. 563800; BD), CD4-APC (S3.5, catalog no. MHCD0405; BD), CD56-BV605 (clone HCD56, catalog no. 318334; Biolegend), and p24/FITC (for intracellular staining) (clone KC57-FITC, catalog no. 6604665; Beckman Coulter).

Cells were analyzed on a BD LSR Fortessa X20 flow cytometer with FACSDiva software (Becton, Dickinson, Mountain View, CA) and analyzed using FlowJo (Tree Star, Inc., Ashland, OR).

### Cytokine analysis.

Supernatants were collected from each well and stored at −20°C until ready for analysis with a IFN-γ enzyme-linked immunosorbent assay (ELISA) kit (catalog no. ENEHIFNG2; Invitrogen) and CXCL-10 U-PLEX human IP-10 assay MSD (catalog no. K151UFK).

### NK cell cytotoxicity assays.

Flow cytometry-based assays were performed using overnight-activated NK cell cultures when K562-GFP (ATCC) cells were used as targets. Pretreated NK cells were washed and incubated in duplicates at 1:1 effector-to-target cell (E/T) ratios for 4 h in 96-well U-bottom plates. After incubation, cells were harvested, washed, and stained for flow cytometry. LIVE/DEAD fixable aqua stain (Invitrogen) was used for assessment of dead K562 cells. In parallel cultures, cells were stained to assess NK cell degranulation and intracellular production of IFN-γ. Anti-PE-CD107a antibody (clone H4A) was added to the cocultures, and after 1 h of incubation, eBioscience protein transport inhibitor cocktail (eBioscience) was added for an additional 3 h. After incubation, cells were harvested, washed, stained with LIVE/DEAD fixable aqua stain (Invitrogen), and surface stained with anti-CD56-BV605 antibody (Biolegend) in PBS plus 3% FBS for 20 min on ice in the dark. Cells were then fixed and permeabilized with Cytofix/Cytoperm buffer (BD Biosciences) and washed with Perm/Wash buffer (BD Biosciences), followed by intracellular staining with anti-TNF-α-APC-Cy7 and anti-IFN-γ-PE-Cy7 antibodies (Biolegend) for 30 min at 4°C. After washing, cells were resuspended in buffer for cytometric analysis. The percentage of direct killing for each treatment was calculated with the following formula: (% of live K562 cells with NK cells) − (% of live K562 cells without NK cells).

The DELFIA (dissociation-enhanced lanthanide fluorescence immunoassay) cell cytotoxicity assay was performed to measure cytotoxicity against cancer cell lines A2780, U87, OCILy1, and OCILy10 according to the manufacturer’s protocol with a few modifications (catalog no. AD0116; PerkinElmer). Adherent cell lines A2780 and U87 were washed 5 times after labeling with BATDA [1,2-bis(2-aminophenoxy)ethane-*N*,*N*,*N*′,*N*′-tetraacetic acid]. OCILy1 and OCILy10 were washed 3 times after labeling with BATDA. The percentage of specific killing was calculated with the following formula: 100 × [(BATDA release with NK cells) − (BATDA release with no NK cells)]/[(maximum BATDA release) − (BATDA release with no NK cells)].

For experiments using HIV_NL43_-infected CD4 T cells as targets, infected cultured central memory CD4 T cells were generated as previously described until day 13 (99), washed, and cocultured overnight with overnight-pretreated NK cells in a 96-well U-bottom plate. Cells were washed and stained for flow cytometry analysis as described above. NK cell-mediated elimination of autologous transmitted/founder (TF) virus CH058-infected cells was evaluated by flow cytometry as previously described (100). Briefly, CD4 T cells were isolated from rested human PBMCs and activated with phytohemagglutinin-L (PHA-L) and rhIL-2. Activated CD4 T cells were then infected with vesicular stomatitis virus G (VSV-G)-pseudotyped transmitted/founder (TF) virus CH058, as described previously (100). Autologous NK cells were isolated from resting PBMCs and treated for 24 h with 100 μM HODHBt or an equivalent volume of DMSO with or without 100 ng/mL of rhIL-15. Twenty-four hours postinfection, infected CD4 T cells were stained with a viability dye (AquaVivid; ThermoFisher Scientific) and a cell proliferation dye (eFluor670; eBioscience) and used as target cells. Autologous purified NK cells, stained with another cellular marker (cell proliferation dye eFluor450; eBioscience), were added at different NK cell/target ratios (1:4, 1:2, and 1:1) in 96-well V-bottom plates (Corning, Corning, NY). The plates were subsequently centrifuged for 1 min at 300 × *g* and incubated at 37°C, 5% CO_2_ for 5 h before being fixed in a 2% PBS–formaldehyde solution. Infected cells were identified by intracellular staining for HIV-1 p24. Samples were acquired on an LSR II flow cytometer (BD Biosciences), and data analysis was performed using FlowJo version X.0.7 (Tree Star). For experiments using CIML NK cells, naive CD4 T cells were activated with anti-CD3/anti-CD28 antibody for 3 days and infected with the HIV_AD8_ molecular clone. At day 7, HIV-infected cells were cocultured overnight with CIML NK cells in a 96-well U-bottom plate. Cells were washed and stained for flow cytometry analysis as described above. In each case, the percentage of direct killing was calculated with the following formula: (% of p24^+^ cells in targets) − (% of p24^+^ cells in targets plus effectors)/(% of p24^+^ cells in targets) by gating on live target cells. For the results shown in Fig. 4, data were normalized as follows (% of p24^+^ cells in targets plus effectors treated with rhIL-15 in combination with either DMSO or HODHBt) − (% of p24^+^ cells in targets plus effectors treated with DMSO or HODHBt alone).

### Statistics.

Statistical analyses were performed using GraphPad Prism 9.0 software (GraphPad Software). The statistical analysis used is indicated in each figure legend. A *P* value of less than 0.05 was considered significant (*, *P* < 0.05; **, *P* < 0.01; and ***, *P* < 0.001). All the data with error bars are presented as mean values ± standard deviations.

### Study approval.

Volunteers 17 years and older at the Gulf Coast Regional Blood Center served as blood participants. White blood cell concentrates (buffy coat) prepared from a single unit of whole blood by centrifugation were purchased.

### Ethics statement.

Written informed consent was obtained from all study participants, and the research adhered to the ethical guidelines of CRCHUM and was reviewed and approved by the CRCHUM Institutional Review Board (Ethics Committee approval number CE 16.164-CA). The research adhered to the standards indicated by the Declaration of Helsinki. All participants were adults and provided informed written consent prior to enrollment, in accordance with Institutional Review Board approval.

### Data availability.

Data have been deposited to NCBI SRA under accession number PRJNA753488.

## References

[1] Chun TW, Engel D, Berrey MM, Shea T, Corey L, Fauci AS. 1998. Early establishment of a pool of latently infected, resting CD4(+) T cells during primary HIV-1 infection. Proc Natl Acad Sci USA 95:8869–8873. 10.1073/pnas.95.15.8869. 9671771 PMC21169

[2] Finzi D, Hermankova M, Pierson T, Carruth LM, Buck C, Chaisson RE, Quinn TC, Chadwick K, Margolick J, Brookmeyer R, Gallant J, Markowitz M, Ho DD, Richman DD, Siliciano RF. 1997. Identification of a reservoir for HIV-1 in patients on highly active antiretroviral therapy. Science 278:1295–1300. 10.1126/science.278.5341.1295. 9360927

[3] Wong JK, Hezareh M, Gunthard HF, Havlir DV, Ignacio CC, Spina CA, Richman DD. 1997. Recovery of replication-competent HIV despite prolonged suppression of plasma viremia. Science 278:1291–1295. 10.1126/science.278.5341.1291. 9360926

[4] Trono D, Van Lint C, Rouzioux C, Verdin E, Barre-Sinoussi F, Chun TW, Chomont N. 2010. HIV persistence and the prospect of long-term drug-free remissions for HIV-infected individuals. Science 329:174–180. 10.1126/science.1191047. 20616270

[5] Flores M, Johnston R. 2016. Curing HIV: moving forward faster. AIDS Res Hum Retroviruses 32:125–128. 10.1089/aid.2016.0004. 26862662 PMC4761812

[6] Barton KM, Burch BD, Soriano-Sarabia N, Margolis DM. 2013. Prospects for treatment of latent HIV. Clin Pharmacol Ther 93:46–56. 10.1038/clpt.2012.202. 23212106 PMC3942883

[7] Richman DD, Margolis DM, Delaney M, Greene WC, Hazuda D, Pomerantz RJ. 2009. The challenge of finding a cure for HIV infection. Science 323:1304–1307. 10.1126/science.1165706. 19265012

[8] Shen L, Siliciano RF. 2008. Viral reservoirs, residual viremia, and the potential of highly active antiretroviral therapy to eradicate HIV infection. J Allergy Clin Immunol 122:22–28. 10.1016/j.jaci.2008.05.033. 18602567 PMC6812482

[9] Spivak AM, Planelles V. 2016. HIV-1 eradication: early trials (and tribulations). Trends Mol Med 22:10–27. 10.1016/j.molmed.2015.11.004. 26691297 PMC5889129

[10] Martinsen JT, Gunst JD, Hojen JF, Tolstrup M, Sogaard OS. 2020. The use of Toll-like receptor agonists in HIV-1 cure strategies. Front Immunol 11:1112. 10.3389/fimmu.2020.01112. 32595636 PMC7300204

[11] Denton PW, Sogaard OS, Tolstrup M. 2019. Impacts of HIV cure interventions on viral reservoirs in tissues. Front Microbiol 10:1956. 10.3389/fmicb.2019.01956. 31497010 PMC6712158

[12] Macedo AB, Novis CL, Bosque A. 2019. Targeting cellular and tissue HIV reservoirs with Toll-like receptor agonists. Front Immunol 10:2450. 10.3389/fimmu.2019.02450. 31681325 PMC6804373

[13] Sung JM, Margolis DM. 2018. HIV persistence on antiretroviral therapy and barriers to a cure. Adv Exp Med Biol 1075:165–185. 10.1007/978-981-13-0484-2_7. 30030793

[14] Board NL, Moskovljevic M, Wu F, Siliciano RF, Siliciano JD. 25 November 2021. Engaging innate immunity in HIV-1 cure strategies. Nat Rev Immunol 10.1038/s41577-021-00649-1. 34824401

[15] Van Lint C, Bouchat S, Marcello A. 2013. HIV-1 transcription and latency: an update. Retrovirology 10:67. 10.1186/1742-4690-10-67. 23803414 PMC3699421

[16] Grau-Exposito J, Luque-Ballesteros L, Navarro J, Curran A, Burgos J, Ribera E, Torrella A, Planas B, Badia R, Martin-Castillo M, Fernandez-Sojo J, Genesca M, Falco V, Buzon MJ. 2019. Latency reversal agents affect differently the latent reservoir present in distinct CD4+ T subpopulations. PLoS Pathog 15:e1007991. 10.1371/journal.ppat.1007991. 31425551 PMC6715238

[17] Walker-Sperling VE, Pohlmeyer CW, Tarwater PM, Blankson JN. 2016. The effect of latency reversal agents on primary CD8+ T cells: implications for shock and kill strategies for human immunodeficiency virus eradication. EBioMedicine 8:217–229. 10.1016/j.ebiom.2016.04.019. 27428432 PMC4919475

[18] Jones RB, Mueller S, O’Connor R, Rimpel K, Sloan DD, Karel D, Wong HC, Jeng EK, Thomas AS, Whitney JB, Lim SY, Kovacs C, Benko E, Karandish S, Huang SH, Buzon MJ, Lichterfeld M, Irrinki A, Murry JP, Tsai A, Yu H, Geleziunas R, Trocha A, Ostrowski MA, Irvine DJ, Walker BD. 2016. A subset of latency-reversing agents expose HIV-infected resting CD4+ T-cells to recognition by cytotoxic T-lymphocytes. PLoS Pathog 12:e1005545. 10.1371/journal.ppat.1005545. 27082643 PMC4833318

[19] Clutton G, Xu Y, Baldoni PL, Mollan KR, Kirchherr J, Newhard W, Cox K, Kuruc JD, Kashuba A, Barnard R, Archin N, Gay CL, Hudgens MG, Margolis DM, Goonetilleke N. 2016. The differential short- and long-term effects of HIV-1 latency-reversing agents on T cell function. Sci Rep 6:30749. 10.1038/srep30749. 27480951 PMC4969750

[20] Cummins NW, Sainski AM, Dai H, Natesampillai S, Pang YP, Bren GD, de Araujo Correia MCM, Sampath R, Rizza SA, O’Brien D, Yao JD, Kaufmann SH, Badley AD. 2016. Prime, shock, and kill: priming CD4 T cells from HIV patients with a BCL-2 antagonist before HIV reactivation reduces HIV reservoir size. J Virol 90:4032–4048. 10.1128/JVI.03179-15. 26842479 PMC4810548

[21] Huang SH, Ren Y, Thomas AS, Chan D, Mueller S, Ward AR, Patel S, Bollard CM, Cruz CR, Karandish S, Truong R, Macedo AB, Bosque A, Kovacs C, Benko E, Piechocka-Trocha A, Wong H, Jeng E, Nixon DF, Ho YC, Siliciano RF, Walker BD, Jones RB. 2018. Latent HIV reservoirs exhibit inherent resistance to elimination by CD8+ T cells. J Clin Invest 128:876–889. 10.1172/JCI97555. 29355843 PMC5785246

[22] Kuo HH, Ahmad R, Lee GQ, Gao C, Chen HR, Ouyang Z, Szucs MJ, Kim D, Tsibris A, Chun TW, Battivelli E, Verdin E, Rosenberg ES, Carr SA, Yu XG, Lichterfeld M. 2018. Anti-apoptotic protein BIRC5 maintains survival of HIV-1-infected CD4(+) T cells. Immunity 48:1183–1194.e5. 10.1016/j.immuni.2018.04.004. 29802019 PMC6013384

[23] Ren Y, Huang SH, Macedo AB, Ward AR, Alberto WDC, Klevorn T, Leyre L, Copertino DC, Mota TM, Chan D, Truong R, Rohwetter T, Zumbo P, Dundar F, Betel D, Kovacs C, Benko E, Bosque A, Jones RB. 2021. Selective BCL-XL antagonists eliminate infected cells from a primary-cell model of HIV latency but not from ex vivo reservoirs. J Virol 95:e0242520. 10.1128/JVI.02425-20. 33980597 PMC8274617

[24] Day CL, Kaufmann DE, Kiepiela P, Brown JA, Moodley ES, Reddy S, Mackey EW, Miller JD, Leslie AJ, DePierres C, Mncube Z, Duraiswamy J, Zhu B, Eichbaum Q, Altfeld M, Wherry EJ, Coovadia HM, Goulder PJ, Klenerman P, Ahmed R, Freeman GJ, Walker BD. 2006. PD-1 expression on HIV-specific T cells is associated with T-cell exhaustion and disease progression. Nature 443:350–354. 10.1038/nature05115. 16921384

[25] Trautmann L, Janbazian L, Chomont N, Said EA, Gimmig S, Bessette B, Boulassel MR, Delwart E, Sepulveda H, Balderas RS, Routy JP, Haddad EK, Sekaly RP. 2006. Upregulation of PD-1 expression on HIV-specific CD8+ T cells leads to reversible immune dysfunction. Nat Med 12:1198–1202. 10.1038/nm1482. 16917489

[26] Miklossy G, Hilliard TS, Turkson J. 2013. Therapeutic modulators of STAT signalling for human diseases. Nat Rev Drug Discov 12:611–629. 10.1038/nrd4088. 23903221 PMC4038293

[27] Zhu X, Marcus WD, Xu W, Lee HI, Han K, Egan JO, Yovandich JL, Rhode PR, Wong HC. 2009. Novel human interleukin-15 agonists. J Immunol 183:3598–3607. 10.4049/jimmunol.0901244. 19710453 PMC2814526

[28] Han KP, Zhu X, Liu B, Jeng E, Kong L, Yovandich JL, Vyas VV, Marcus WD, Chavaillaz PA, Romero CA, Rhode PR, Wong HC. 2011. IL-15:IL-15 receptor alpha superagonist complex: high-level co-expression in recombinant mammalian cells, purification and characterization. Cytokine 56:804–810. 10.1016/j.cyto.2011.09.028. 22019703 PMC3221918

[29] Miller JS, Davis ZB, Helgeson E, Reilly C, Thorkelson A, Anderson J, Lima NS, Jorstad S, Hart GT, Lee JH, Safrit JT, Wong H, Cooley S, Gharu L, Chung H, Soon-Shiong P, Dobrowolski C, Fletcher CV, Karn J, Douek DC, Schacker TW. 2022. Safety and virologic impact of the IL-15 superagonist N-803 in people living with HIV: a phase 1 trial. Nat Med 28:392–400. 10.1038/s41591-021-01651-9. 35102335

[30] McBrien JB, Mavigner M, Franchitti L, Smith SA, White E, Tharp GK, Walum H, Busman-Sahay K, Aguilera-Sandoval CR, Thayer WO, Spagnuolo RA, Kovarova M, Wahl A, Cervasi B, Margolis DM, Vanderford TH, Carnathan DG, Paiardini M, Lifson JD, Lee JH, Safrit JT, Bosinger SE, Estes JD, Derdeyn CA, Garcia JV, Kulpa DA, Chahroudi A, Silvestri G. 2020. Robust and persistent reactivation of SIV and HIV by N-803 and depletion of CD8(+) cells. Nature 578:154–159. 10.1038/s41586-020-1946-0. 31969705 PMC7580846

[31] McBrien JB, Wong AKH, White E, Carnathan DG, Lee JH, Safrit JT, Vanderford TH, Paiardini M, Chahroudi A, Silvestri G. 2020. Combination of CD8beta depletion and interleukin-15 superagonist N-803 induces virus reactivation in simian-human immunodeficiency virus-infected, long-term ART-treated rhesus macaques. J Virol 94:e00755-20. 10.1128/JVI.00755-20. 32669328 PMC7495383

[32] Garrido C, Abad-Fernandez M, Tuyishime M, Pollara JJ, Ferrari G, Soriano-Sarabia N, Margolis DM, Kirchhoff F. 2018. Interleukin-15-stimulated natural killer cells clear HIV-1-infected cells following latency reversal ex vivo. J Virol 92:e00235-18. 10.1128/JVI.00235-18. 29593039 PMC5974478

[33] Seay K, Church C, Zheng JH, Deneroff K, Ochsenbauer C, Kappes JC, Liu B, Jeng EK, Wong HC, Goldstein H. 2015. In vivo activation of human NK cells by treatment with an interleukin-15 superagonist potently inhibits acute in vivo HIV-1 infection in humanized mice. J Virol 89:6264–6274. 10.1128/JVI.00563-15. 25833053 PMC4474292

[34] Younes SA, Freeman ML, Mudd JC, Shive CL, Reynaldi A, Panigrahi S, Estes JD, Deleage C, Lucero C, Anderson J, Schacker TW, Davenport MP, McCune JM, Hunt PW, Lee SA, Serrano-Villar S, Debernardo RL, Jacobson JM, Canaday DH, Sekaly RP, Rodriguez B, Sieg SF, Lederman MM. 2016. IL-15 promotes activation and expansion of CD8+ T cells in HIV-1 infection. J Clin Invest 126:2745–2756. 10.1172/JCI85996. 27322062 PMC4922693

[35] Webb GM, Molden J, Busman-Sahay K, Abdulhaqq S, Wu HL, Weber WC, Bateman KB, Reed JS, Northrup M, Maier N, Tanaka S, Gao L, Davey B, Carpenter BL, Axthelm MK, Stanton JJ, Smedley J, Greene JM, Safrit JT, Estes JD, Skinner PJ, Sacha JB. 2020. The human IL-15 superagonist N-803 promotes migration of virus-specific CD8+ T and NK cells to B cell follicles but does not reverse latency in ART-suppressed, SHIV-infected macaques. PLoS Pathog 16:e1008339. 10.1371/journal.ppat.1008339. 32163523 PMC7093032

[36] Webb GM, Li S, Mwakalundwa G, Folkvord JM, Greene JM, Reed JS, Stanton JJ, Legasse AW, Hobbs T, Martin LD, Park BS, Whitney JB, Jeng EK, Wong HC, Nixon DF, Jones RB, Connick E, Skinner PJ, Sacha JB. 2018. The human IL-15 superagonist ALT-803 directs SIV-specific CD8(+) T cells into B-cell follicles. Blood Adv 2:76–84. 10.1182/bloodadvances.2017012971. 29365313 PMC5787870

[37] Kubo M, Hanada T, Yoshimura A. 2003. Suppressors of cytokine signaling and immunity. Nat Immunol 4:1169–1176. 10.1038/ni1012. 14639467

[38] Croker BA, Kiu H, Nicholson SE. 2008. SOCS regulation of the JAK/STAT signalling pathway. Semin Cell Dev Biol 19:414–422. 10.1016/j.semcdb.2008.07.010. 18708154 PMC2597703

[39] Tanaka T, Soriano MA, Grusby MJ. 2005. SLIM is a nuclear ubiquitin E3 ligase that negatively regulates STAT signaling. Immunity 22:729–736. 10.1016/j.immuni.2005.04.008. 15963787

[40] Chen Y, Wen R, Yang S, Schuman J, Zhang EE, Yi T, Feng GS, Wang D. 2003. Identification of Shp-2 as a Stat5A phosphatase. J Biol Chem 278:16520–16527. 10.1074/jbc.M210572200. 12615921

[41] Rogers RS, Horvath CM, Matunis MJ. 2003. SUMO modification of STAT1 and its role in PIAS-mediated inhibition of gene activation. J Biol Chem 278:30091–30097. 10.1074/jbc.M301344200. 12764129

[42] Van Nguyen T, Angkasekwinai P, Dou H, Lin FM, Lu LS, Cheng J, Chin YE, Dong C, Yeh ET. 2012. SUMO-specific protease 1 is critical for early lymphoid development through regulation of STAT5 activation. Mol Cell 45:210–221. 10.1016/j.molcel.2011.12.026. 22284677 PMC3269036

[43] Bosque A, Nilson KA, Macedo AB, Spivak AM, Archin NM, Van Wagoner RM, Martins LJ, Novis CL, Szaniawski MA, Ireland CM, Margolis DM, Price DH, Planelles V. 2017. Benzotriazoles reactivate latent HIV-1 through inactivation of STAT5 SUMOylation. Cell Rep 18:1324–1334. 10.1016/j.celrep.2017.01.022. 28147284 PMC5461578

[44] Sorensen ES, Macedo AB, Resop RS, Howard JN, Nell R, Sarabia I, Newman D, Ren Y, Jones RB, Planelles V, Spivak AM, Bosque A. 2020. Structure-activity relationship analysis of benzotriazine analogues as HIV-1 latency-reversing agents. Antimicrob Agents Chemother 64:e00888-20. 10.1128/AAC.00888-20. 32482680 PMC7526807

[45] Huntington ND, Vosshenrich CA, Di Santo JP. 2007. Developmental pathways that generate natural-killer-cell diversity in mice and humans. Nat Rev Immunol 7:703–714. 10.1038/nri2154. 17717540

[46] Cerwenka A, Lanier LL. 2016. Natural killer cell memory in infection, inflammation and cancer. Nat Rev Immunol 16:112–123. 10.1038/nri.2015.9. 26806484

[47] Glimcher LH, Townsend MJ, Sullivan BM, Lord GM. 2004. Recent developments in the transcriptional regulation of cytolytic effector cells. Nat Rev Immunol 4:900–911. 10.1038/nri1490. 15516969

[48] Lozzio BB, Lozzio CB. 1979. Properties and usefulness of the original K-562 human myelogenous leukemia cell line. Leuk Res 3:363–370. 10.1016/0145-2126(79)90033-x. 95026

[49] Bliss CI. 1939. The toxicity of poisons applied jointly. Ann Appl Biol 26:585–615. 10.1111/j.1744-7348.1939.tb06990.x.

[50] Alter G, Heckerman D, Schneidewind A, Fadda L, Kadie CM, Carlson JM, Oniangue-Ndza C, Martin M, Li B, Khakoo SI, Carrington M, Allen TM, Altfeld M. 2011. HIV-1 adaptation to NK-cell-mediated immune pressure. Nature 476:96–100. 10.1038/nature10237. 21814282 PMC3194000

[51] Cohen GB, Gandhi RT, Davis DM, Mandelboim O, Chen BK, Strominger JL, Baltimore D. 1999. The selective downregulation of class I major histocompatibility complex proteins by HIV-1 protects HIV-infected cells from NK cells. Immunity 10:661–671. 10.1016/S1074-7613(00)80065-5. 10403641

[52] Fauci AS, Mavilio D, Kottilil S. 2005. NK cells in HIV infection: paradigm for protection or targets for ambush. Nat Rev Immunol 5:835–843. 10.1038/nri1711. 16239902

[53] Cooper MA, Elliott JM, Keyel PA, Yang L, Carrero JA, Yokoyama WM. 2009. Cytokine-induced memory-like natural killer cells. Proc Natl Acad Sci USA 106:1915–1919. 10.1073/pnas.0813192106. 19181844 PMC2644138

[54] O’Leary JG, Goodarzi M, Drayton DL, von Andrian UH. 2006. T cell- and B cell-independent adaptive immunity mediated by natural killer cells. Nat Immunol 7:507–516. 10.1038/ni1332. 16617337

[55] Sun JC, Beilke JN, Lanier LL. 2009. Adaptive immune features of natural killer cells. Nature 457:557–561. 10.1038/nature07665. 19136945 PMC2674434

[56] Romee R, Rosario M, Berrien-Elliott MM, Wagner JA, Jewell BA, Schappe T, Leong JW, Abdel-Latif S, Schneider SE, Willey S, Neal CC, Yu L, Oh ST, Lee YS, Mulder A, Claas F, Cooper MA, Fehniger TA. 2016. Cytokine-induced memory-like natural killer cells exhibit enhanced responses against myeloid leukemia. Sci Transl Med 8:357ra123. 10.1126/scitranslmed.aaf2341. PMC5436500 27655849

[57] Romee R, Schneider SE, Leong JW, Chase JM, Keppel CR, Sullivan RP, Cooper MA, Fehniger TA. 2012. Cytokine activation induces human memory-like NK cells. Blood 120:4751–4760. 10.1182/blood-2012-04-419283. 22983442 PMC3520618

[58] Barbulescu K, Becker C, Schlaak JF, Schmitt E, Meyer Zum Buschenfelde KH, Neurath MF. 1998. IL-12 and IL-18 differentially regulate the transcriptional activity of the human IFN-gamma promoter in primary CD4+ T lymphocytes. J Immunol 160:3642–3647. 9558063

[59] Nagler A, Lanier LL, Cwirla S, Phillips JH. 1989. Comparative studies of human FcRIII-positive and negative natural killer cells. J Immunol 143:3183–3191. 2530273

[60] Jacobs R, Hintzen G, Kemper A, Beul K, Kempf S, Behrens G, Sykora KW, Schmidt RE. 2001. CD56bright cells differ in their KIR repertoire and cytotoxic features from CD56dim NK cells. Eur J Immunol 31:3121–3127. 10.1002/1521-4141(2001010)31:10<3121::AID-IMMU3121>3.0.CO;2-4. 11592089

[61] Huot N, Jacquelin B, Garcia-Tellez T, Rascle P, Ploquin MJ, Madec Y, Reeves RK, Derreudre-Bosquet N, Muller-Trutwin M. 2017. Natural killer cells migrate into and control simian immunodeficiency virus replication in lymph node follicles in African green monkeys. Nat Med 23:1277–1286. 10.1038/nm.4421. 29035370 PMC6362838

[62] Rerks-Ngarm S, Pitisuttithum P, Nitayaphan S, Kaewkungwal J, Chiu J, Paris R, Premsri N, Namwat C, de Souza M, Adams E, Benenson M, Gurunathan S, Tartaglia J, McNeil JG, Francis DP, Stablein D, Birx DL, Chunsuttiwat S, Khamboonruang C, Thongcharoen P, Robb ML, Michael NL, Kunasol P, Kim JH, MOPH-TAVEG Investigators. 2009. Vaccination with ALVAC and AIDSVAX to prevent HIV-1 infection in Thailand. N Engl J Med 361:2209–2220. 10.1056/NEJMoa0908492. 19843557

[63] Bradley T, Pollara J, Santra S, Vandergrift N, Pittala S, Bailey-Kellogg C, Shen X, Parks R, Goodman D, Eaton A, Balachandran H, Mach LV, Saunders KO, Weiner JA, Scearce R, Sutherland LL, Phogat S, Tartaglia J, Reed SG, Hu SL, Theis JF, Pinter A, Montefiori DC, Kepler TB, Peachman KK, Rao M, Michael NL, Suscovich TJ, Alter G, Ackerman ME, Moody MA, Liao HX, Tomaras G, Ferrari G, Korber BT, Haynes BF. 2017. Pentavalent HIV-1 vaccine protects against simian-human immunodeficiency virus challenge. Nat Commun 8:15711. 10.1038/ncomms15711. 28593989 PMC5472724

[64] Saez-Cirion A, Bacchus C, Hocqueloux L, Avettand-Fenoel V, Girault I, Lecuroux C, Potard V, Versmisse P, Melard A, Prazuck T, Descours B, Guergnon J, Viard JP, Boufassa F, Lambotte O, Goujard C, Meyer L, Costagliola D, Venet A, Pancino G, Autran B, Rouzioux C, ANRS VISCNTI Study Group. 2013. Post-treatment HIV-1 controllers with a long-term virological remission after the interruption of early initiated antiretroviral therapy ANRS VISCONTI Study. PLoS Pathog 9:e1003211. 10.1371/journal.ppat.1003211. 23516360 PMC3597518

[65] Gotthardt D, Sexl V. 2016. STATs in NK-cells: the good, the bad, and the ugly. Front Immunol 7:694. 10.3389/fimmu.2016.00694. 28149296 PMC5241313

[66] Eckelhart E, Warsch W, Zebedin E, Simma O, Stoiber D, Kolbe T, Rulicke T, Mueller M, Casanova E, Sexl V. 2011. A novel Ncr1-Cre mouse reveals the essential role of STAT5 for NK-cell survival and development. Blood 117:1565–1573. 10.1182/blood-2010-06-291633. 21127177

[67] Imada K, Bloom ET, Nakajima H, Horvath-Arcidiacono JA, Udy GB, Davey HW, Leonard WJ. 1998. Stat5b is essential for natural killer cell-mediated proliferation and cytolytic activity. J Exp Med 188:2067–2074. 10.1084/jem.188.11.2067. 9841920 PMC2212377

[68] Gotthardt D, Putz EM, Grundschober E, Prchal-Murphy M, Straka E, Kudweis P, Heller G, Bago-Horvath Z, Witalisz-Siepracka A, Cumaraswamy AA, Gunning PT, Strobl B, Muller M, Moriggl R, Stockmann C, Sexl V. 2016. STAT5 is a key regulator in NK cells and acts as a molecular switch from tumor surveillance to tumor promotion. Cancer Discov 6:414–429. 10.1158/2159-8290.CD-15-0732. 26873347

[69] Zuniga EI, Macal M, Lewis GM, Harker JA. 2015. Innate and adaptive immune regulation during chronic viral infections. Annu Rev Virol 2:573–597. 10.1146/annurev-virology-100114-055226. 26958929 PMC4785831

[70] Nabatanzi R, Bayigga L, Cose S, Rowland-Jones S, Canderan G, Joloba M, Nakanjako D. 2019. Aberrant natural killer (NK) cell activation and dysfunction among ART-treated HIV-infected adults in an African cohort. Clin Immunol 201:55–60. 10.1016/j.clim.2019.02.010. 30817998 PMC6448528

[71] Alter G, Altfeld M. 2009. NK cells in HIV-1 infection: evidence for their role in the control of HIV-1 infection. J Intern Med 265:29–42. 10.1111/j.1365-2796.2008.02045.x. 19093958 PMC2842208

[72] Chehimi J, Azzoni L, Farabaugh M, Creer SA, Tomescu C, Hancock A, Mackiewicz A, D’Alessandro L, Ghanekar S, Foulkes AS, Mounzer K, Kostman J, Montaner LJ. 2007. Baseline viral load and immune activation determine the extent of reconstitution of innate immune effectors in HIV-1-infected subjects undergoing antiretroviral treatment. J Immunol 179:2642–2650. 10.4049/jimmunol.179.4.2642. 17675528

[73] Giuliani E, Vassena L, Di Cesare S, Malagnino V, Desimio MG, Andreoni M, Barnaba V, Doria M. 2017. NK cells of HIV-1-infected patients with poor CD4(+) T-cell reconstitution despite suppressive HAART show reduced IFN-gamma production and high frequency of autoreactive CD56(bright) cells. Immunol Lett 190:185–193. 10.1016/j.imlet.2017.08.014. 28826739

[74] Hernandez-Ramirez RU, Shiels MS, Dubrow R, Engels EA. 2017. Cancer risk in HIV-infected people in the USA from 1996 to 2012: a population-based, registry-linkage study. Lancet HIV 4:e495–e504. 10.1016/S2352-3018(17)30125-X. 28803888 PMC5669995

[75] Rubinstein PG, Aboulafia DM, Zloza A. 2014. Malignancies in HIV/AIDS: from epidemiology to therapeutic challenges. AIDS 28:453–465. 10.1097/QAD.0000000000000071. 24401642 PMC4501859

[76] Barry KC, Hsu J, Broz ML, Cueto FJ, Binnewies M, Combes AJ, Nelson AE, Loo K, Kumar R, Rosenblum MD, Alvarado MD, Wolf DM, Bogunovic D, Bhardwaj N, Daud AI, Ha PK, Ryan WR, Pollack JL, Samad B, Asthana S, Chan V, Krummel MF. 2018. A natural killer-dendritic cell axis defines checkpoint therapy-responsive tumor microenvironments. Nat Med 24:1178–1191. 10.1038/s41591-018-0085-8. 29942093 PMC6475503

[77] Chockley PJ, Chen J, Chen G, Beer DG, Standiford TJ, Keshamouni VG. 2018. Epithelial-mesenchymal transition leads to NK cell-mediated metastasis-specific immunosurveillance in lung cancer. J Clin Invest 128:1384–1396. 10.1172/JCI97611. 29324443 PMC5873856

[78] Cursons J, Souza-Fonseca-Guimaraes F, Foroutan M, Anderson A, Hollande F, Hediyeh-Zadeh S, Behren A, Huntington ND, Davis MJ. 2019. A gene signature predicting natural killer cell infiltration and improved survival in melanoma patients. Cancer Immunol Res 7:1162–1174. 10.1158/2326-6066.CIR-18-0500. 31088844

[79] Nath PR, Pal-Nath D, Mandal A, Cam MC, Schwartz AL, Roberts DD. 2019. Natural killer cell recruitment and activation are regulated by CD47 expression in the tumor microenvironment. Cancer Immunol Res 7:1547–1561. 10.1158/2326-6066.CIR-18-0367. 31362997 PMC6726576

[80] Takanami I, Takeuchi K, Giga M. 2001. The prognostic value of natural killer cell infiltration in resected pulmonary adenocarcinoma. J Thorac Cardiovasc Surg 121:1058–1063. 10.1067/mtc.2001.113026. 11385371

[81] Wu M, Mei F, Liu W, Jiang J. 2020. Comprehensive characterization of tumor infiltrating natural killer cells and clinical significance in hepatocellular carcinoma based on gene expression profiles. Biomed Pharmacother 121:109637. 10.1016/j.biopha.2019.109637. 31810126

[82] Gang M, Marin ND, Wong P, Neal CC, Marsala L, Foster M, Schappe T, Meng W, Tran J, Schaettler M, Davila M, Gao F, Cashen AF, Bartlett NL, Mehta-Shah N, Kahl BS, Kim MY, Cooper ML, DiPersio JF, Berrien-Elliott MM, Fehniger TA. 2020. CAR-modified memory-like NK cells exhibit potent responses to NK-resistant lymphomas. Blood 136:2308–2318. 10.1182/blood.2020006619. 32614951 PMC7702478

[83] Kweon S, Phan MT, Chun S, Yu H, Kim J, Kim S, Lee J, Ali AK, Lee SH, Kim SK, Doh J, Cho D. 2019. Expansion of human NK cells using K562 cells expressing OX40 ligand and short exposure to IL-21. Front Immunol 10:879. 10.3389/fimmu.2019.00879. 31105701 PMC6491902

[84] Ojo EO, Sharma AA, Liu R, Moreton S, Checkley-Luttge MA, Gupta K, Lee G, Lee DA, Otegbeye F, Sekaly RP, de Lima M, Wald DN. 2019. Membrane bound IL-21 based NK cell feeder cells drive robust expansion and metabolic activation of NK cells. Sci Rep 9:14916. 10.1038/s41598-019-51287-6. 31624330 PMC6797802

[85] Phan MT, Lee SH, Kim SK, Cho D. 2016. Expansion of NK cells using genetically engineered K562 feeder cells. Methods Mol Biol 1441:167–174. 10.1007/978-1-4939-3684-7_14. 27177665

[86] Paust S, von Andrian UH. 2011. Natural killer cell memory. Nat Immunol 12:500–508. 10.1038/ni.2032. 21739673

[87] Sun JC, Lanier LL. 2011. Versatility in NK cell memory. Immunol Cell Biol 89:327–329. 10.1038/icb.2010.162. 21173784 PMC4406234

[88] Gondois-Rey F, Cheret A, Mallet F, Bidaut G, Granjeaud S, Lecuroux C, Ploquin M, Muller-Trutwin M, Rouzioux C, Avettand-Fenoel V, De Maria A, Pialoux G, Goujard C, Meyer L, Olive D. 2017. A mature NK profile at the time of HIV primary infection is associated with an early response to cART. Front Immunol 8:54. 10.3389/fimmu.2017.00054. 28239376 PMC5300971

[89] Gondois-Rey F, Cheret A, Granjeaud S, Mallet F, Bidaut G, Lecuroux C, Ploquin M, Muller-Trutwin M, Rouzioux C, Avettand-Fenoel V, Moretta A, Pialoux G, Goujard C, Meyer L, Olive D. 2017. NKG2C(+) memory-like NK cells contribute to the control of HIV viremia during primary infection: Optiprim-ANRS 147. Clin Transl Immunol 6:e150. 10.1038/cti.2017.22. PMC5539415 28791125

[90] Reeves RK, Li H, Jost S, Blass E, Li H, Schafer JL, Varner V, Manickam C, Eslamizar L, Altfeld M, von Andrian UH, Barouch DH. 2015. Antigen-specific NK cell memory in rhesus macaques. Nat Immunol 16:927–932. 10.1038/ni.3227. 26193080 PMC4545390

[91] Wang Y, Lifshitz L, Gellatly K, Vinton CL, Busman-Sahay K, McCauley S, Vangala P, Kim K, Derr A, Jaiswal S, Kucukural A, McDonel P, Hunt PW, Greenough T, Houghton J, Somsouk M, Estes JD, Brenchley JM, Garber M, Deeks SG, Luban J. 2020. HIV-1-induced cytokines deplete homeostatic innate lymphoid cells and expand TCF7-dependent memory NK cells. Nat Immunol 21:274–286. 10.1038/s41590-020-0593-9. 32066947 PMC7044076

[92] Leong JW, Chase JM, Romee R, Schneider SE, Sullivan RP, Cooper MA, Fehniger TA. 2014. Preactivation with IL-12, IL-15, and IL-18 induces CD25 and a functional high-affinity IL-2 receptor on human cytokine-induced memory-like natural killer cells. Biol Blood Marrow Transplant 20:463–473. 10.1016/j.bbmt.2014.01.006. 24434782 PMC3959288

[93] Smith SL, Kennedy PR, Stacey KB, Worboys JD, Yarwood A, Seo S, Solloa EH, Mistretta B, Chatterjee SS, Gunaratne P, Allette K, Wang YC, Smith ML, Sebra R, Mace EM, Horowitz A, Thomson W, Martin P, Eyre S, Davis DM. 2020. Diversity of peripheral blood human NK cells identified by single-cell RNA sequencing. Blood Adv 4:1388–1406. 10.1182/bloodadvances.2019000699. 32271902 PMC7160259

[94] Kay HD, Fagnani R, Bonnard GD. 1979. Cytotoxicity against the K562 erythroleukemia cell line by human natural killer (NK) cells which do not bear free Fc receptors for IgG. Int J Cancer 24:141–150. 10.1002/ijc.2910240204. 290570

[95] Ewels P, Magnusson M, Lundin S, Kaller M. 2016. MultiQC: summarize analysis results for multiple tools and samples in a single report. Bioinformatics 32:3047–3048. 10.1093/bioinformatics/btw354. 27312411 PMC5039924

[96] Bolger AM, Lohse M, Usadel B. 2014. Trimmomatic: a flexible trimmer for Illumina sequence data. Bioinformatics 30:2114–2120. 10.1093/bioinformatics/btu170. 24695404 PMC4103590

[97] Love MI, Huber W, Anders S. 2014. Moderated estimation of fold change and dispersion for RNA-seq data with DESeq2. Genome Biol 15:550. 10.1186/s13059-014-0550-8. 25516281 PMC4302049

[98] Jassal B, Matthews L, Viteri G, Gong C, Lorente P, Fabregat A, Sidiropoulos K, Cook J, Gillespie M, Haw R, Loney F, May B, Milacic M, Rothfels K, Sevilla C, Shamovsky V, Shorser S, Varusai T, Weiser J, Wu G, Stein L, Hermjakob H, D’Eustachio P. 2020. The reactome pathway knowledgebase. Nucleic Acids Res 48:D498–D503. 31691815 10.1093/nar/gkz1031 PMC7145712

[99] Macedo AB, Resop RS, Martins LJ, Szaniawski MA, Sorensen ES, Spivak AM, Nixon DF, Jones RB, Planelles V, Bosque A. 2018. Influence of biological sex, age, and HIV status in an in vitro primary cell model of HIV latency using a CXCR4 tropic virus. AIDS Res Hum Retroviruses 34:769–777. 10.1089/AID.2018.0098. 29926732 PMC6152854

[100] Prevost J, Pickering S, Mumby MJ, Medjahed H, Gendron-Lepage G, Delgado GG, Dirk BS, Dikeakos JD, Sturzel CM, Sauter D, Kirchhoff F, Bibollet-Ruche F, Hahn BH, Dube M, Kaufmann DE, Neil SJD, Finzi A, Richard J. 2019. Upregulation of BST-2 by type I interferons reduces the capacity of Vpu To protect HIV-1-infected cells from NK cell responses. mBio 10:e01113-19. 10.1128/mBio.01113-19. 31213558 PMC6581860

