# Supplementary material for: The HIV Latency Reversal Agent HODHBt Enhances NK Cell Effector and Memory-Like Functions by Increasing Interleukin-15-Mediated STAT Activation
Source: J Virol. 2022 Jul 14;96(15):e00372-22. doi: 10.1128/jvi.00372-22 (PMC9364794; doi:10.1128/jvi.00372-22)
Supplement: Supplemental file 5 — Captions to Tables S1 to S4. Download jvi.00372-22-s0005.pdf, PDF file, 0.02 MB [file jvi.00372-22-s0005.pdf]

Supplemental Tables

Supplemental Table 1. RNA-seq analysis of all genes from NK cell stimulated with DMSO, IL-15, HODHBt, and IL-15 plus HODHBt from 4 donors. F= female and M= male.

Supplemental Table 2. Differentially expressed genes of NK cells stimulated with IL-15, HODHBt, and IL-15 plus HODHBt relative to DMSO control. Values are shown as mean from 4 donors

Supplemental Table 3. Differentially expressed genes between IL-15 and IL-15 plus HODHBt treatment. Values are shown as mean from 4 donors.

Supplemental Table 4. Reactome pathway analysis. Top 25 pathways differentially regulated between IL-15 and IL-15 plus HODHBt treatment.
